# Calcium and Superoxide-Mediated Pathways Converge to Induce Nitric Oxide-Dependent Apoptosis in *Mycobacterium fortuitum*-Infected Fish Macrophages

**DOI:** 10.1371/journal.pone.0146554

**Published:** 2016-01-11

**Authors:** Debika Datta, Preeti Khatri, Chaitali Banerjee, Ambika Singh, Ramavatar Meena, Dhira Rani Saha, Rajagopal Raman, Paulraj Rajamani, Abhijit Mitra, Shibnath Mazumder

**Affiliations:** 1 Immunobiology Laboratory, Department of Zoology, University of Delhi, Delhi, India; 2 Gut Biology Laboratory, Department of Zoology, University of Delhi, Delhi, India; 3 School of Environmental Sciences, Jawaharlal Nehru University, Delhi, India; 4 Microscopy Laboratory, National Institute of Cholera and Enteric Diseases, Kolkata, India; 5 Genome Analysis Laboratory, Animal Division, Indian Veterinary Research Institute, Izatnagar, Bareilly, India; IISER-TVM, INDIA

## Abstract

*Mycobacterium fortuitum* causes ‘mycobacteriosis’ in wide range of hosts although the mechanisms remain largely unknown. Here we demonstrate the role of calcium (Ca^+2^)-signalling cascade on *M*. *fortuitum-*induced apoptosis in headkidney macrophages (HKM) of *Clarias* sp. *M*. *fortuitum* could trigger intracellular-Ca^+2^ influx leading to the activation of calmodulin (CaM), protein kinase C alpha (PKCα) and Calmodulin kinase II gamma (CaMKII*g*). Gene silencing and inhibitor studies established the role of CaM in *M*. *fortuitum* pathogenesis. We noted that CaMKII*g* activation is regulated by CaM as well as PKCα-dependent superoxide anions. This is altogether first report of oxidised CaMKII*g* in mycobacterial infections. Our studies with targeted-siRNA and pharmacological inhibitors implicate CaMKII*g* to be pro-apoptotic and critical for the activation of extra-cellular signal regulated kinase 1/2 (ERK1/2). Inhibiting the ERK1/2 pathway attenuated nitric oxide synthase 2 (NOS2)-induced nitric oxide (NO) production. Conversely, inhibiting the NOS2-NO axis by specific-siRNA and inhibitors down-regulated ERK1/2 activation suggesting the crosstalk between ERK1/2 and NO is essential for pathogenesis induced by the bacterium. Silencing the NOS2-NO axis enhanced intracellular bacterial survival and attenuated caspase-8 mediated activation of caspase-3 in the infected HKM. Our findings unveil hitherto unknown mechanism of *M*. *fortuitum* pathogenesis. We propose that *M*. *fortuitum* triggers intracellular Ca^+2^ elevations resulting in CaM activation and PKCα-mediated superoxide generation. The cascade converges in common pathway mediated by CaMKII*g* resulting in the activation of ERK1/2-NOS2 axis. The crosstalk between ERK1/2 and NO shifts the balance in favour of caspase dependent apoptosis of *M*. *fortuitum*-infected HKM.

## Introduction

*Mycobacterium fortuitum*, a rapidly growing and atypical mycobacteria, is one of the causative agents of piscine-mycobacteriosis. *M*. *fortuitum* is pathogen of concern not only because of its impact on aquaculture and zoonosis [1] but also due to increased reports from immuno-compromised individuals [2] and occurrence of multidrug resistant strains [3]. Despite its wide range of infectivity, reports detailing the molecular pathogenesis and virulent attributes of *M*. *fortuitum* are obscure.

Calcium (Ca^+2^) is a versatile intracellular messenger that regulates different cellular functions. An increase in cytosolic Ca^+2^ influxes can trigger apoptosis in several cell systems. *M*. *tuberculosis*-induced apoptosis through persistent Ca^+2^ influx in infected macrophages is reported earlier [4]. The major effect of cytosolic Ca^+2^ influx is the increased expression of 17-kDa protein calmodulin (CaM) that functions as the predominant Ca^+2^ sensor in eukaryotic cells. CaM has four distinct structural motifs known as the ‘E-F hand’. Each E-F hand consists of an N-terminal helix (the E helix), a centrally located Ca^+2^ coordinating loop and a C-terminal helix (the F helix). The binding of Ca^+2^ induces conformational changes in CaM releasing considerable free energy and exposing critical hydrophobic patches on the surface of CaM that binds to complementary sites present on numerous effector proteins [5]. The involvement of Ca^+2^-CaM pathway in *M*. *bovis* BCG infection has been reported [6]. An important downstream effector is calmodulin-dependent protein kinase II (CaMKII), a multifunctional Ser/Thr kinase. Binding of Ca^+2^-CaM relieves auto inhibition, resulting in inter subunit phosphorylation and activation of CaMKII. The Ca^+2^-CaM-CaMKII pathway has been implicated in the activation of other signalling pathways including mitogen activated protein kinase (MAPK) during mycobacterial pathogenesis [7]. There are several isoforms of CaMKII and the pro-apoptotic role of the gamma-isoform (CaMKII*g*) on macrophage apoptosis has been increasingly reported [8].

Protein kinase C (PKC) comprises a family of Ser/Thr kinases that perform myriad of functions including immune signalling [9]. On the basis of structure and cofactor requirement PKC are classified into conventional-PKC (cPKC), novel-PKC (nPKC) and atypical-PKC (aPKC). PKC activation as host containment or defence phenomenon is well implicated in mycobacterial infections [10]. It has been suggested that cPKC signalling is integral to ROS generation and the activation of MAPK family proteins, though the mechanisms remain poorly understood [11]. The generation of ROS constitutes an important macrophagic response to mycobacteria by impacting several intracellular signalling pathways with pathophysiological implications [12]. Recent reports suggest that mycobacterial virulence factors alter intracellular Ca^+2^-levels leading to enhanced ROS production and subsequent macrophage apoptosis [13].

Mitogen activated protein kinase (MAPK) represents an evolutionarily conserved kinase cascade which acts as focal point in the regulation of diverse cellular functions including cell proliferation or death [14]. Extra cellular signal regulated kinase (ERK 1/2) is an important component of the MAPK family and there are reports suggesting the kinase contributing to pathogenesis induced by several pathogenic mycobacteria [6, 14, 15]. Among the several important genes that are up-regulated by ERK 1/2 in mycobacterial infections NOS2 is important [16]. Nitric oxide induced *via* the NOS2 pathway inhibits the growth of mycobacteria and is reported to be critical for clearing the pathogen from infected mice [17, 18]. However, the role of NO in case of atypical mycobacterial pathogenesis is inconclusive [19]. NO induces its pro-apoptotic effect through the activation of caspase-8 [20]. Pathological conditions lead to different outcomes, of which apoptosis has been greatly studied with respect to mycobacterial infections [21]. Although, caspase-mediated apoptosis is considered to be the classical pathway there are reports suggesting the initiation of the death program could also be caspase-independent in mycobacterial infection [22, 23]. Caspase-mediated apoptosis occurs through two distinct pathways, the extrinsic or caspase-8 and intrinsic or caspase-9 pathway which often cross-talk and have been implicated in mycobacterial infections [21]. The final step in the caspase cascade is the activation of executioner caspase or caspase-3. The implication of apoptosis in mycobacterial pathogenesis is a matter of speculation. On one hand, there are studies documenting apoptosis limits mycobacterial spread and infection [24, 25]. Results from other groups [26, 27] also suggest that the apoptosing macrophages might act as Trojan horse in the dissemination of mycobacteria to unsuspecting macrophages. It has also been suggested that virulent mycobacteria induce necrosis [28] or necroptosis [29] rather than apoptosis of infected macrophages.

It is important to note that information pertaining to mycobacterial pathogenesis is primarily based on mammalian models against typical mycobacterial pathogens. There is little information on the pathogenicity induced by atypical mycobacteria like *M*. *fortuitum*. Fish serves as natural hosts for *M*. *fortuitum*. They possess an elaborate innate immune arm and fish macrophages are critical for countering different pathogens [30]. In the present study, we explored the mechanisms of *M*. *fortuitum* pathology using macrophages isolated from head kidney (HK) or anterior kidney from *Clarias* sp. The HK is an important lymphoid organ in fish and rich source of macrophages [31]. In recent years, work from our laboratory has successfully established that HKM are inherently phagocytic and serve as an alternate model to study bacterial infection [8, 30, 32].

Here, we sought to study the role of Ca^+2^-dependent signalling molecules on *M*. *fortuitum-*induced pathology. Our study demonstrated the primal role of CaM and PKCα-mediated ROS with CaMKII*g* acting as platform wherein the two pathways integrate initiating cascade of events leading to extrinsic pathway mediated apoptosis of *M*. *fortuitum-*infected HKM.

## Material and Methods

### Ethics Statement

Animal experiments described in this study were approved by the Animal Ethics Committee, University of Delhi (DU/ZOOL/IAEC-R/2013/34) and carried out in accordance with the protocols approved by The Prevention of Cruelty to Animals Act, Govt. of India.

### Bacterial strains and growth conditions

*Mycobacterium fortuitum* (Strain 993) was purchased from Microbial Type Culture Collection and Gene Bank (MTCC), Chandigarh, India. For infection studies, the bacteria were grown to midlog phase (120 h) in Middlebrook 7H9 broth (Himedia) at 30°C supplemented with 0.05% Tween-80, 0.50% glycerol and 100 μg/mL ampicillin. The stocks were maintained at -80°C in 10% glycerol as well as Lowenstein Jensen media (Himedia) at 4°C for further use. The anti-microbial profile suggested the strain to be amikacin sensitive.

### Isolation of HKM and infection with *M*. *fortuitum*

Catfish *(Clarias sp)* 100–150 g obtained from local firm were maintained in 50-L glass tanks (2–3 fish per tank) under natural photoperiod. Prior to initiating the study fish were acclimatized to the laboratory conditions for 15 d. Fish were fed boiled chicken liver *ad libitum*. The water quality and fish health were monitored regularly during the entire span of the study [8]. The headkidney macrophages (HKM) were isolated from head-kidney according to the standard protocol reported earlier [8, 32]. The HKM were washed in antibiotic-free RPMI supplemented with 10% FBS (complete RPMI) and infected with *M*. *fortuitum* at a multiplicity of infection (MOI) of 1: 10 (HKM: bacteria). The number of HKM used for different experiments are mentioned in corresponding section. A short spin of 5 min was given to facilitate bacteria-HKM interactions, the cells distributed in 6 well tissue culture plates and incubated for 4 h at 30°C. Subsequently, amikacin (50 μg/mL, HiMedia) was added and the cells further incubated for 1 h to kill the extra-cellular bacteria. The concentration of amikacin effectively killed extra-cellular bacteria without affecting HKM viability (data not shown). Finally, the infected HKM were washed and re-suspended in complete RPMI containing amikacin (5 μg/mL) and incubated at 30°C for further studies.

### Inhibitors used

Intracellular Ca^+2^ chelator [1, 2-Bis (2-aminophenoxy) ethane-N,N,N′,N′-tetraacetic acid tetrakis (acetoxymethyl ester), BAPTA/AM, 5 μM)], NADPH Oxidase inhibitor (Diphenyleneiodonium chloride, DPI, 10 μM), calmodulin inhibitor (Calmidazolium chloride, CMZ, 10 nM), pan-PKC inhibitor (Chelerythrin chloride, CC, 5 μM), PKCα inhibitor (Gö6976, 5 μM), Raf-1 kinase inhibitor (GW5074, 10 μM), inducible nitric oxide synthase inhibitor (aminoguanidine hemisulfate, AMG, 1 mM and *N*_ω_-Nitro-L-arginine methyl ester hydrochloride, L-NAME, 1 mM), were purchased from Sigma. NADPH Oxidase inhibitor (Apocynin, APO, 100 μM) was purchased from Calbiochem. Competitive inhibitor of CaMKII (KN-93, 10 μM) and its structural analogue (KN-92, 10 μM) were purchased from Cayman and Calbiochem respectively. MEK1/2 inhibitor (PD98059, 10 μM) was purchased from Selleck chemicals. The MEK-ERK1/2 inhibitor (U0126, 20 μM) was from Promega. Pan-caspase inhibitor (Z-VAD-FMK, 10 μM), caspase-8 inhibitor (Z-IETD-FMK, 10 μM,) and caspase-3 inhibitor (Z-DEVD-FMK, 10 μM) were purchased from Biovision. The HKM were pre-treated with the specific inhibitors for 1 h prior to infection with *M*. *fortuitum*. The concentration of different inhibitors was based on their specificity and cytotoxicity. The HKM treated with the indicated concentrations of the inhibitors remained as viable as control HKM at all time points as determined by the trypan blue (0.4%) dye exclusion method (data not shown) and were maintained during the entire course of the experiment.

### siRNA Transfection

The siRNA transfection was carried out using HiPerFect Transfection Reagent (Qiagen) as described earlier [8]. Briefly, the siRNA-HiPerFect complex (5 μl each) was added gently to Opti-MEM (Invitrogen), incubated for 20 min then added to the HKM cultures maintained in Opti-MEM. The HKM-siRNA complex was incubated overnight at 30°C with 5% CO_2,_ washed, placed in complete-RPMI and infected with *M*. *fortuitum* as mentioned above. Targeted gene knock down was confirmed by Real-Time PCR, protein and apoptosis assays. Five nano mole each of targeted [(CaM, SENSE-5’-CCAUUACGACCAAAGAGUU-3’ & ANTISENSE-5’-AACUCUUUGGUCGUAAUGG-3’); (CaMKII*g*, SENSE-5’- GGACAUUUGGGCUUGUGGA-3’ & ANTISENSE-5’-UCCACAAGCCCAAAUGUCC-3’) [8], (NOS2, SENSE-5’-CGCUACAACAUUCUUGAGA-3’ & ANTISENSE-5’-UCUCAAGAAUGUUGUAGCG-3’)] and siRNA Universal negative CONTROL (Sigma) were used for this study.

### Apoptosis study

HKM pre-treated or transfected with or without indicated concentrations of respective inhibitors and targeted or scrambled siRNAs were infected with or without *M*. *fortuitum* and apoptotic changes studied at 24 h p.i.

#### (i)TUNEL assay

Control and infected HKM (1×10^6^) were fixed in 1% paraformaldehyde solution then washed and Terminal transferase dUTP nick end labelling assay (TUNEL assay) was performed using *In situ* apoptosis detection kit (Apop Tag Plus Fluorescein In Situ Detection Kit, Chemicon, Millipore) according to the manufactures protocol. In brief, the TdT enzyme was added to the samples and incubated for 30 mins at 30°C following which the stop buffer was added. After washing with PBS anti-digoxigenin-fluorescein conjugate was added to the samples and incubated at 30°C in dark. Nuclear stain was done with DAPI (1 μg/mL, Sigma) and the cells were visualized by confocal microscope (× 40 oil immersion, Nikon Eclipse A1Rsi-T*i*E-300). HKM were incubated with apoptosis inducer staurosporine (STS, 1 μM, Sigma) for 6 h as positive control for the assay.

#### (ii)Hoechst 33342

Control and infected HKM (1×10^6^) were washed, fixed with 3.7% paraformaldehyde solution at room temperature, stained with Hoechst 33342 (2 μg / mL, Sigma) and visualized under fluorescence microscope (×40, Nikon Eclipse 400) within 30 mins of adding the stain. Total 100 cells were studied in each field and three fields were observed to determine the percentage of apoptotic HKM [8]. HKM were incubated with apoptosis inducer staurosporine (STS, 1 μM, Sigma) for 6 h as positive control for the assay.

#### (iii)AnnexinV^FITC^ & Propidium Iodide (AV-PI)

The AV^FITC^-PI staining was performed following the manufacturer’s instructions (BD-Pharmingen). Briefly, control and infected HKM (1×10^6^) were washed and stained with AV^FITC^ -PI mixture and analysed under fluorescence microscope (× 40, Nikon Eclipse 400) within 30 mins of adding the dyes. Hundred HKM were studied in each field and three such fields were included to determine the percentage of apoptotic HKM [8]. HKM were incubated with apoptosis inducer staurosporine (STS, 1 μM, Sigma) for 6 h as positive control for the assay.

#### (iv)TEM

Control and infected HKM (1×10^7^) were washed, fixed with 2.5% glutaraldehyde (Polaron, Biorad) in 0.1 M phosphate buffer (pH 7.4). The fixed HKM were treated with 1% phosphate buffered OsO4 (Sigma), dehydrated through graded series of ethanol and propylene oxide (Merck) and embedded in Epon 812 (TAAB). Ultra-thin sections (60 nm, Ultramicrotome, Leica) were placed on nickel grids (Sigma), stained with uranyl acetate (BDH) and lead citrate (Polaron) and examined under Tecnai 12 Bio-twin transmission electron microscope (FEI, 80 kV) [33].

### Measurement of intracellular-Ca^+2^ levels

The intracellular-Ca^+2^ was measured using the Fluo-4 (Fluo-4 Direct Calcium Assay Kit, Life Technologies) following the manufacturer’s protocol. Briefly, the HKM (1×10^5^) pre-treated with or without indicated inhibitors were infected with *M*. *fortuitum* for the indicated time intervals. The HKM were washed, loaded with Fluo-4 and changes in the fluorescent intensity recorded using microplate reader at 485 nm excitation and 520 nm emissions respectively (BioTek, Synergy HT) [34].

### Measurement of superoxide anion levels

The production of ROS (superoxide radicals) was detected and quantified by fluorimetric assays using DHE (Invitrogen, 5 μM). Briefly, the HKM (1×10^6^) uninfected or infected with *M*. *fortuitum* for indicated time intervals were washed and incubated with DHE for 15 mins at 30°C. The changes in fluorescence intensity were quantified by microplate reader at excitation and emission at 520 and 580 nm respectively.

In a parallel study, the HKM (1×10^6^) were pre-treated with or without indicated inhibitors followed by infection with *M*. *fortuitum* washed and incubated with DHE for 15 mins at 30°C. The changes in fluorescence intensity were quantified by FACS (BD Accuri).

### RNA extraction and cDNA synthesis

The RNA was isolated from HKM (2×10^7^) control or transfected separately with or without targeted or scrambled siRNA and infected with *M*. *fortuitum* at indicated time point p.i. using TRIZOL (Sigma). From 1 μg of DNase treated (RNase-free) RNA the cDNA was prepared using first strand cDNA synthesis kit as per manufacturer’s instructions (MBI Fermentas).

### Cloning, Amplification, Sequencing of NOS2

To identify the NOS2 gene in *Clarias* sp the degenerate primers were designed (Table 1). The cDNA was amplified; the product extracted using HiPura gel extraction kit (Himedia), cloned into pGEM-T EASY vector (Promega) and sequenced (Macrogen). The sequence obtained (Table 2) were aligned to nBLAST and submitted to NCBI database (Accession No. KF956810).

**Table 1:** Degenerate primers for NOS2.

NOS2-Degenerate primers	
Forward Primers	Reverse Primers
FP1 5’-TAYGCTGGCTAYCAGATG—3’	RP1 5’-CTGYTGCCAGAARCTKCGGAA- 3’
FP2 5’-GGYTGGTACATGGGCACMGA—3’	RP2 5’-ATGRGCAAAGGCRCAGAACYG- 3’
	RP3 5’-CATCTCCTGGTGRAASACRGG- 3’
R = A/G; Y = C/T; K = G/T, M = A/C	

**Table 2:** NOS2 gene sequence.

> NOS2 (Accession No. KF956810)
GGTTGGTACATGGGCACAGAGATTGGAGCAAGGGACTTCTGTGATCCTCAGCGCTACAACATTCTTGAGAAAGTTGGACGCTGTATGGGGTTGGATACACACAAGCTTTCATCGCTATGGAAGGATGAAGCTCTAGTTGCTGTCAATGTTGCAGTGATTCACAGTTTTCAGAAAAATAAAGTGACCATCACAGACCACCACACTGCCACAGAGTCCTTCATGAAGTACATGGAGACAGAATTGCGCCTGCGTGGTGGCTGTCCTGCCGACTGGGTTTGGCTGGTACCTCCTATGTCTGGCTCTCTGACCCCCGTCTTCCACCAGGAGATG

### Real-time PCR

The quantification of CaM, CaMKII*g* and NOS2 were done using SYBR green PCR Master Mix (Applied Biosystems) by Real-Time PCR (ABI ViiA, Applied Biosystems) as described earlier [8]. The gene specific real-time primers have been listed in Table 3. The expression of different genes were analysed by comparative ΔΔC_T_ method wherein β-actin was taken as the internal control (endogenous control) and uninfected HKM (0h) was used as the calibrator.

**Table 3:** Real-time primers for qPCR.

Gene	Real-Time Primers	Size	Accession No.
CaM	FP: 5’-AAGATGGAGATGGCACCATTA-3’	150	KF892532
	RP: 5’-TGGTCAGGAACTCTGGGAAG-3’		
CaMKII*g*	FP: 5’-TTGTTGACATCTGGGCATGT-3’	111	KF892533
	RP: 5’- CATAAGCTCCGCTTTGATCT -3’		
NOS2	FP: 5’-GACCATCACAGACCACCACA-3’	114	KF956810
	RP: 5’-GACATAGGAGGTACCAGCCAA-3’		
β-actin	FP: 5’- CGAGCAGGAGATGGGAACC-3’	102	AF057040
	RP: 5’-CAACGGAAACGCTCATTGC-3’		

### PKC activity assay

The PKC activity was quantified using PKC kinase activity assay kit (Enzo Life Sciences) following the manufacturer’s protocol with chemicals supplied with the kit. The wells provided with the kit were pre-coated with PKC substrate which gets phosphorylated by the active PKC. The HKM (1×10^6^) pre-treated with or without inhibitors were infected with *M*. *fortuitum*, collected at indicated time p.i. and re-suspended in chilled lysis buffer containing protease inhibitors. The lysates were added to the appropriate wells followed by the addition of ATP to initiate the reaction. The assay was developed with tetramethylbenzidine substrate (TMB) and the relative change in PKC phosphotransferase activity plotted from the OD (450 nm) values obtained.

### CaM & CaMKII*g* Assay

CaM and CaMKII*g* assays were performed following the manufacturer’s protocol (USCN Life Science Inc.) with chemicals supplied with the kit. Briefly, HKM (1×10^6^) pre-incubated or transfected with or without inhibitors or siRNAs were infected with or without *M*. *fortuitum*. The HKM were collected at indicated time p.i. and re-suspended in chilled lysis buffer containing protease inhibitors. For the quantitative detection of CaM and CaMKII*g*, the cell lysates were harvested and the assays performed in plates pre-coated with biotin-conjugated antibody specific to CaM or CaMKII*g* [8]. The amount of CaM and CaMKII*g* were interpolated from their respective standard curves obtained by plotting the O.D. (450 nm) of the standards.

### ERK 1 /2 Assay

The total ERK 1/2 and pERK 1/2 were measured using specific kits (Enzo Life Sciences). The HKM (1×10^6^) pre-treated or transfected with or without indicated inhibitors or siRNAs respectively followed by *M*. *fortuitum* infection. At 24 h p.i. the cell lysates collected and the assays performed in microtiter plates pre-coated with antibody specific to total ERK 1/2 and pERK 1/2 respectively [8]. The assays were developed with TMB substrate, reaction terminated by addition of stop solution and absorbance read at 450 nm using a microplate reader. The amount of total and pERK 1/2 were interpolated from their respective standard curves obtained by plotting the O.D. of the standards.

### Immunocytochemistry of NOS2

To detect NOS2 expression, the HKM (5×10^6^) were pre-incubated with or without different inhibitors or transfected with specific siRNA and infected with *M*. *fortuitum* as mentioned earlier. At 24 h p.i. the cells were fixed in 3% paraformaldehyde at room temperature for 30 min and subsequently incubated with PBS containing BSA (2 mg/mL, blocking solution) and saponin (0.2 mg/mL, permeabilization solution) for 1 h at room temperature. After washing the HKM were incubated with mouse anti-NOS2 primary antibody (1: 200) overnight at 4°C. Next day, the HKM were washed and stained with FITC-conjugated anti-mouse secondary antibody (1: 250) for 3 h at 30°C. The nuclear staining was done by adding DAPI (1 μg/mL, Sigma). The cells were visualized by confocal microscope (× 40 oil immersion, Nikon Eclipse A1Rsi-T*i*E-300).

### Nitric oxide assay

The HKM (1×10^6^) pre-treated separately with or without indicated concentrations of inhibitors, siRNA (targeted/scrambled) for indicated time periods were infected with or without *M*. *fortuitum*. Sodium nitroprusside (SNP, 2 mM, Enzo Life Sc) was used as positive control in the assay. The cell free supernatants were collected at indicated time p.i., mixed with equal volume of Griess reagent (Invitrogen-Molecular Probes) and incubated at 30°C for 10 min. The absorbance was read at 540 nm in microplate reader and the amount of nitrite generated was calculated from the NaNO_2_ standard curve.

### Caspase-8 and Caspase-3 assay

The caspase-3 and -8 activities were detected using specific assay kits from Biovision and GenScript respectively following the manufacturer’s instruction using chemicals supplied with the kit. The HKM (1×10^6^) pre-incubated with or without inhibitors were infected with *M*. *fortuitum* as described earlier were collected 24 h p.i., lysed and the supernatant obtained (50 μl) incubated with caspase-8 or -3 specific substrates in reaction buffer for 5 h at 30°C. The *p*NA light emission was quantified using a microtiter plate reader at 405 nm and the relative fold changes of caspase-8, caspase-3 plotted.

### Quantification of intracellular *M*. *fortuitum* growth

The HKM (1×10^6^) pre-treated with or without indicated inhibitors or transfected with specific siRNA were infected with *M*. *fortuitum* as described earlier. The cells were harvested at indicated time intervals lysed with 0.1% Triton X-100, serially diluted and plated on middlebrook 7H11 agar containing ampicillin (100 μg/ mL). The number of viable bacteria (CFU) was enumerated following incubation at 30°C.

### Statistical analysis

Paired t- test was done to calculate the statistical analysis of the data in between the groups of uninfected, infected and infected followed by pre-treatment with indicated inhibitors or transfection with specific siRNA. p< 0.05 was considered as the minimum significant level.

## Results

### *M*. *fortuitum*-induced HKM apoptosis is caspase dependent

The HKM were infected with *M*. *fortuitum* at MOI 1: 10 and 24 h post infection (p.i.) we noted significant cell death as evident from trypan blue staining. Microscopic observations suggested characteristic changes in HKM *viz*., cell shrinkage, increased granularity, cell rounding, cell aggregation and detachment from the culture plates unlike the uniform monolayer with elongated morphology of uninfected HKM (data not shown).

**Fig 1:**
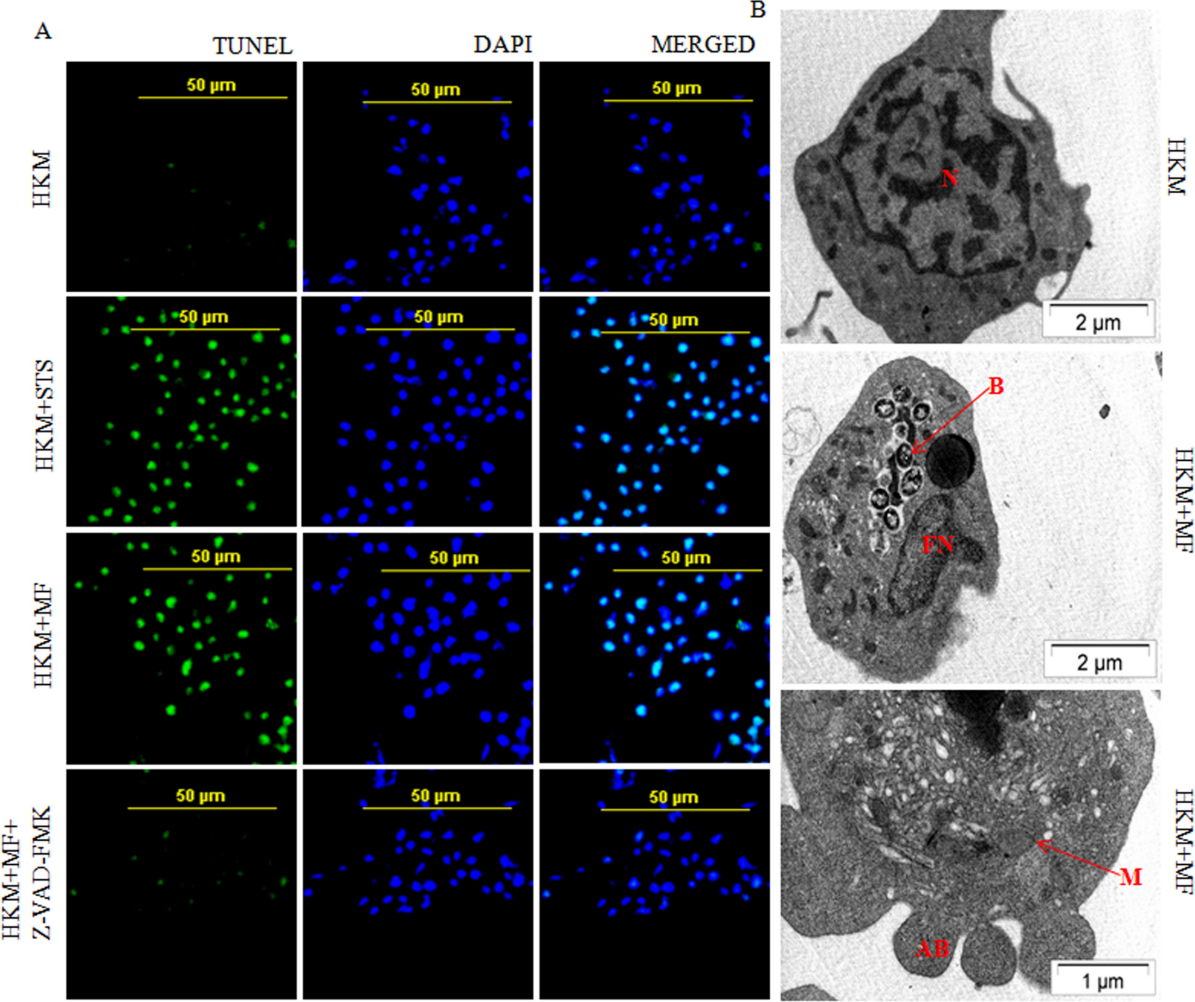
*M*. *fortuitum* induces caspase dependent HKM apoptosis. (A) TUNEL assay of uninfected, STS treated (positive control), *M*. *fortuitum*-infected with or without Z-VAD-FMK pre-treated HKM. The HKM were observed under confocal microscope (×40). (B) TEM analysis of uninfected and *M*. *fortuitum*-infected HKM. All the experiments were performed 24 h p.i. STS treated HKM were analysed 6 h post incubation. The images are representative of three independent experiments. N, nucleus; M, mitochondrion; FN, fragmented nucleus; B, bacteria; AB, apoptotic bodies. HKM, control head kidney macrophage; HKM+STS, HKM treated with STS; HKM+MF, HKM infected with *M*. *fortuitum*; HKM+MF+Z-VAD-FMK, HKM pre-treated with Z-VAD-FMK followed by *M*. *fortuitum* infection.

We were interested to determine the nature of cell death induced by *M*. *fortuitum*. Terminal deoxynucleotidyl transferase (TdT) dUTP nick-End labeling (TUNEL) assay has been designed to detect apoptotic cells that undergo extensive DNA degradation. We observed significant TUNEL positive HKM (45.23% ± 1.5, p<0.05) due to infection with *M*. *fortuitum* compared to the uninfected control cells 5.01% ± 2.1 (Fig 1A) which suggested that *M*. *fortuitum* infection lead to the macrophage apoptosis. Significant number of TUNEL positive HKM (53.55 ± 2.1; p<0.05) were noted following treatment with STS (positive control) for 6 h.

**Fig 2:**
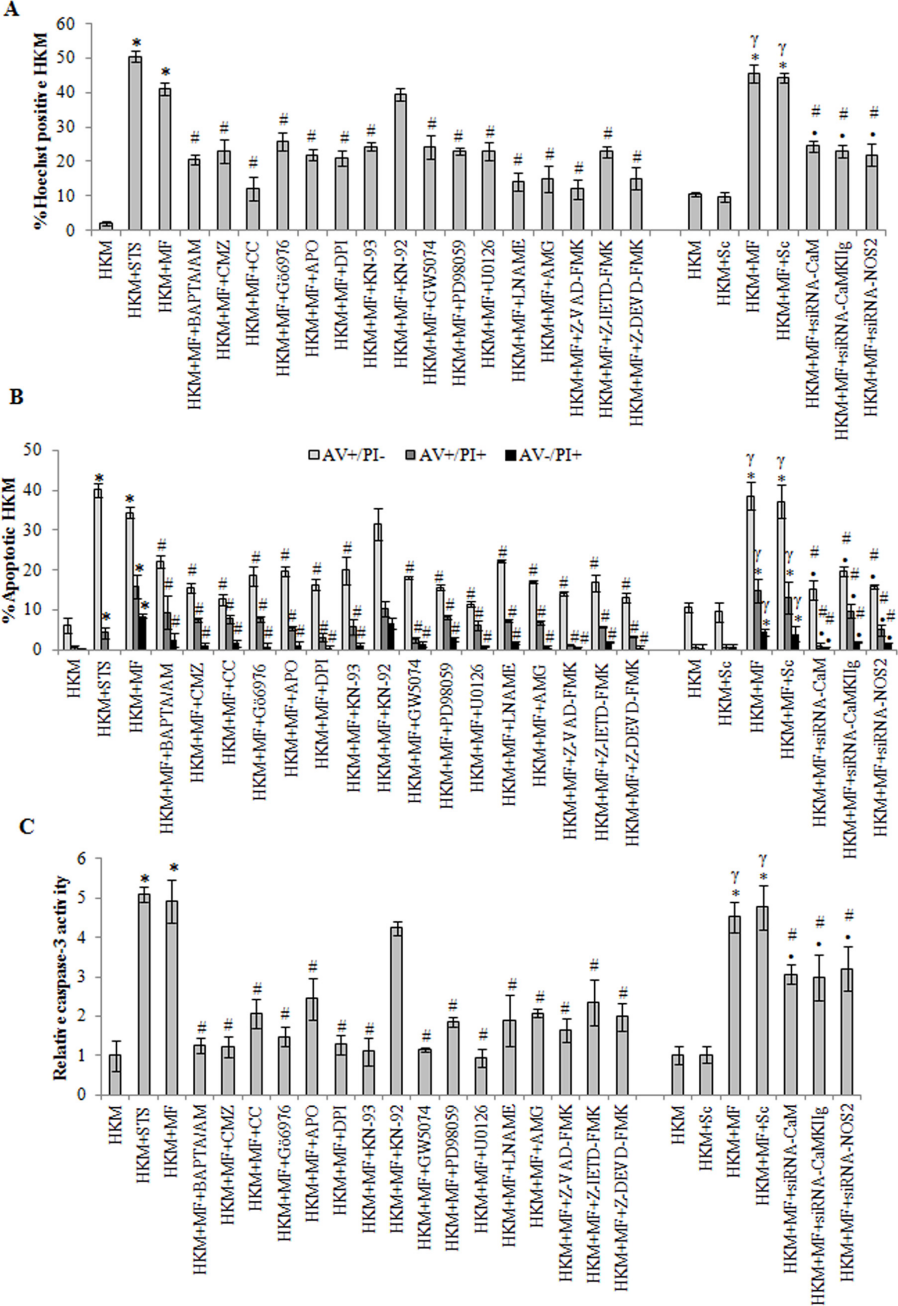
The involvement of pro-apoptotic intermediates in *M*. *fortuitum* infection. HKM were pre-treated separately with indicated inhibitors or transfected with specific siRNAs prior to *M*. *fortuitum* infection and at 24 h p.i. apoptosis measured by (A) Hoechst 33342 staining (B) AV/PI staining and (C) relative caspase-3 activity. STS treated HKM were analysed 6 h post incubation. Vertical bars represent mean ± SE (n = 3).*P<0.05, compared to HKM; ^**γ**^*P*<0.05, compared to HKM+Sc; ^#^*P*<0.05, compared to HKM+MF; ^**•**^*P*<0.05, compared to HKM+MF+Sc. HKM, control head kidney macrophage; HKM+STS, HKM treated with STS (positive control); HKM+Sc, HKM transfected with scrambled siRNA; HKM+MF, HKM infected with *M*. *fortuitum*; HKM+MF+Sc, HKM transfected with scrambled siRNA infected with *M*. *fortuitum*; HKM+MF+siRNA-CaM, HKM transfected with siRNA-CaM infected with *M*. *fortuitum*; HKM+MF+siRNA-CaMKII*g*, HKM transfected with siRNA-CaMKII*g* infected with *M*. *fortuitum*; HKM+MF+siRNA-NOS2, HKM transfected with siRNA-NOS2 infected with *M*. *fortuitum*; HKM+MF+BAPTA/AM, HKM+MF+CMZ, HKM+MF+CC, HKM+MF+Gö6976, HKM+MF+APO, HKM+MF+DPI, HKM+MF+KN-93, HKM+MF+KN-92, HKM+MF+GW5074, HKM+MF+PD98059, HKM+MF+U0126, HKM+MF+LNAME, HKM+MF+AMG, HKM+MF+Z-VAD-FMK, HKM+MF+Z-IETD-FMK, HKM+MF+Z-DEVD-FMK, HKM pre-treated with BAPTA/AM, CMZ, CC, Gö6976, APO, DPI, KN-93, KN-92, GW5074, PD98059, U0126, LNAME, AMG, Z-VAD-FMK, Z-IETD-FMK or Z-DEVD-FMK respectively and infected with *M*. *fortuitum*.

For further proof the control and *M*. *fortuitum*-infected HKM were collected 24 h p.i., stained with Hoechst 33342 and observed under fluorescence microscope (Fig 2A and S1 Fig). Significant number of HKM infected with *M*. *fortuitum* exhibited condensed and intensely stained fragmented nuclei (41.17% ± 1.71, p<0.05), characteristic of apoptosis whereas the control HKM appeared diffusely stained with few Hoechst positive cells (2.10% ± 0.59) suggesting onset of apoptotic events in *M*. *fortuitum*-infected HKM. Treatment with STS (positive control) for 6 h induced significant HKM apoptosis (50.55% ± 1.5; p < 0.05).

In parallel study HKM were collected 24 h p.i., stained with AV/PI and analysed by fluorescence microscopy (Fig 2B and S1 Fig). We observed significant number of AV^+^PI^-^ *M*. *fortuitum*-infected HKM (34.33% ± 1.37 p<0.05) compared to the uninfected control HKM (6.11% ± 1.91) which further emphasised apoptosis as the outcome of *M*. *fortuitum*-induced pathogenicity in HKM. STS (positive control) treated HKM for 6 h showed significant presence of AV^+^PI^-^ cells (40.03% ± 1.6, p < 0.05).

Finally, we used TEM to study *M*. *fortuitum*-induced ultra-structural changes. TEM demonstrated distinct morphological changes that included pseudopodial retraction, plasma membrane blebbing, nuclear fragmentation, disintegration of mitochondria, condensation of chromatin and vacuolization, characteristic of apoptosis. Control HKM exhibited extended pseudopodia, regular nucleus with diffused chromatin (Fig 1B). Collectively, our results confirmed that HKM-cytopathy induced by *M*. *fortuitum* is apoptotic in nature.

Macrophage apoptosis induced by mycobacterial pathogens has been observed to be both caspase-dependent and -independent. To address this, we used the pan caspase inhibitor Z-VAD-FMK and observed that pre-treatment with the inhibitor significantly reduced *M*. *fortuitum*-induced HKM apoptosis (Figs 1A, 2A and 2B) confirming the role of caspase in *M*. *fortuitum* pathogenesis. The next step was to assay executioner caspase activation and we noted significant increase in caspase-3 activity in *M*. *fortuitum-*infected HKM (Fig 2C). Pre-treatment with the caspase-3 inhibitor Z-DEVD-FMK resulted in significant inhibition in caspase-3 activity (Fig 2C), with concomitant reduction in HKM apoptosis (Fig 2A and 2B). Treatment with STS (positive control) induced significant caspase-3 activity in HKM (Fig 2C).

The role of caspase inhibitors on intracellular growth of *M*. *fortuitum* was studied by dilution plating of HKM pre-treated with or without Z-VAD-FMK and Z-DEVD-FMK. We observed that the intracellular survival of *M*. *fortuitum* was significantly improved in presence of both Z-VAD-FMK and Z-DEVD-FMK (S2 Fig). The caspase inhibitors did not influence bacterial growth *per se* when added to 7H9 broth or complete-RPMI (data not shown). Together, these observations confirmed that *M*. *fortuitum-*induced HKM apoptosis is caspase-dependent.

### *M*. *fortuitum* induces intracellular Ca^+2^-surge in HKM

**Fig 3:**
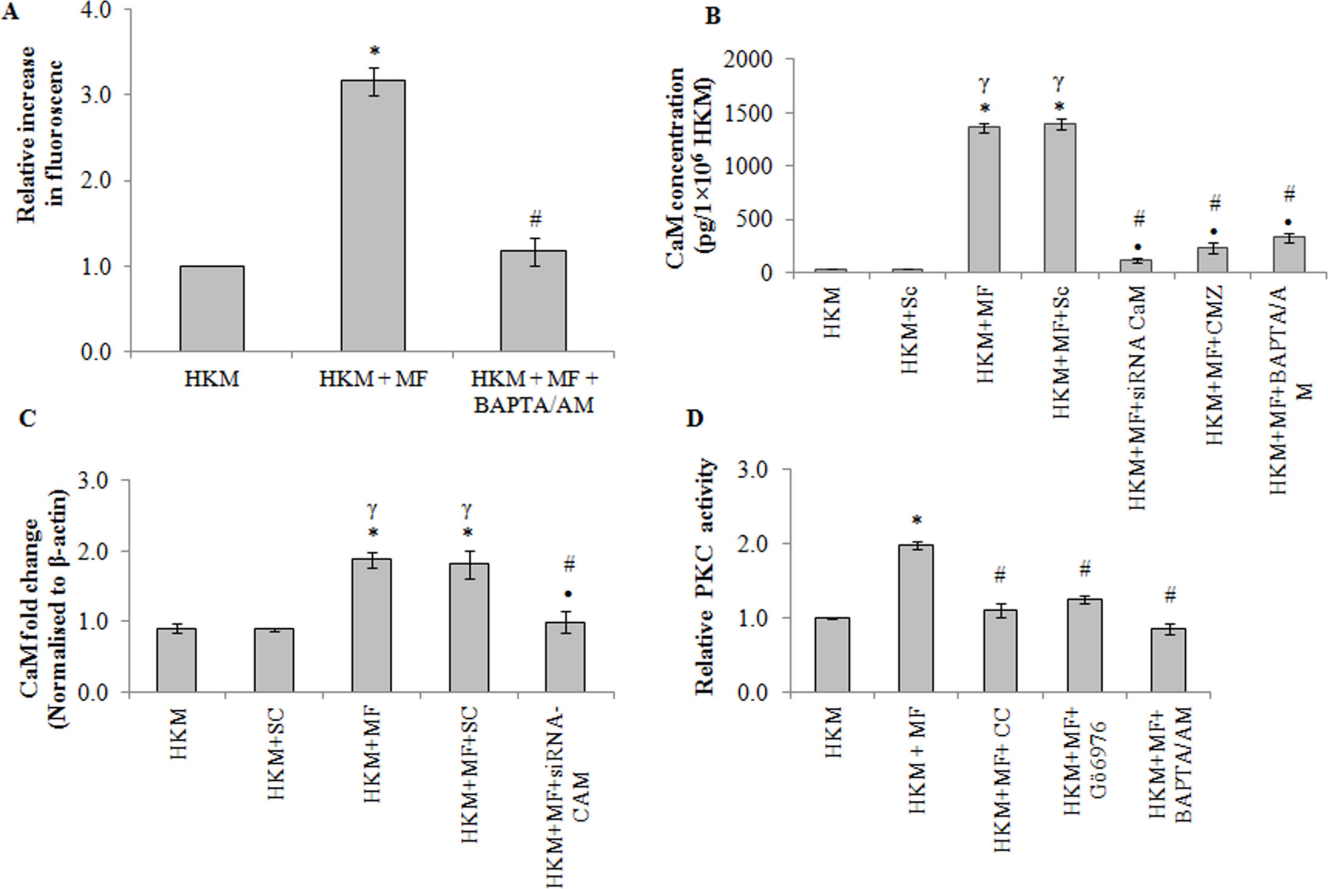
*M*. *fortuitum* alters intracellular Ca^+2^ homeostasis leading to activation of CaM and PKC-α. (A) HKM were pre-treated with or without BAPTA/AM followed by *M*. *fortuitum* infection and cytosolic Ca^+2^ elevation was measured using Fluo-4 at 1 h of infection. (B) HKM transfected with indicated siRNA or pre-treated separately with indicated inhibitors and at 4 h p.i. CaM-protein expression detected by EIA. (C) Fold changes in CaM-mRNA expression was determined in HKM transfected with specific siRNA or non-targeted siRNA followed by *M*. *fortuitum* infection 4 h p.i by qPCR. (D) HKM were pre-treated separately with or without indicated inhibitors and PKC activity determined at 2 h p.i. Vertical bars represent mean ± SE (n = 3).*P<0.05, compared to HKM; ^γ^*P*<0.05, compared to HKM+Sc; ^#^*P*<0.05, compared to HKM+MF; ^•^*P*<0.05, compared to HKM+MF+Sc. HKM, control head kidney macrophage; HKM+Sc, HKM transfected with scrambled siRNA; HKM+MF, HKM infected with *M*. *fortuitum*; HKM+MF+Sc, HKM transfected with scrambled siRNA infected with *M*. *fortuitum*; HKM+MF+siRNA-CaM, HKM transfected with siRNA-CaM infected with *M*. *fortuitum*; HKM+MF+BAPTA/AM, HKM+MF+CMZ, HKM+MF+CC, HKM+MF+Gö6976, HKM were pre treated with BAPTA/AM, CMZ, CC, Gö6979 respectively followed by infection with *M*. *fortuitum*.

The role of Ca^+2^ has been implicated in the pathogenicity of mycobacterial infections. We were interested to study *M*. *fortuitum*-induced alterations in cytosolic Ca^+2^ levels and determine the role of altered Ca^+2^ homeostasis on HKM apoptosis. As a first step, Fluo-4 was used to study *M*. *fortuitum*-induced alterations in cytosolic Ca^+2^ levels. It was observed that *M*. *fortuitum*-infection led to gradual increase in Ca^+2^ levels as early as 15 mins of infection reaching its peak by 60 min; thereafter the levels gradually declined reaching basal levels 2 h p.i. (S3 Fig). Pre-treatment with BAPTA/AM down regulated the Ca^+2^ surge (Fig 3A), inhibited caspase-3 activation (Fig 2C) and attenuated HKM apoptosis (Fig 2A and 2B). In continuation to this, we noted that pre-treatment with BAPTA/AM enhanced intra-cellular bacterial survival (S2 Fig) Together these results highlighted the critical role of intracellular Ca^+2^ on *M*. *fortuitum*-induced HKM apoptosis.

### Cytosolic Ca^+2^ elevation lead to CaM over-expression

On establishing the key role of Ca^+2^ we set out to identify the Ca^+2^-dependent pathways activated consequent to *M*. *fortuitum* infection. Calmodulin (CaM) being an evolutionary conserved Ca^+2^ sensor was our prime target. *M*. *fortuitum* infection led to significant increase in CaM mRNA expression with maximum levels recorded 4 h p.i. (S3 Fig) thereafter, gradually declined reaching the basal levels by 24 h p.i. Maximum CaM protein was also detected 4 h p.i. (S3 Fig). Hence, we selected 4 h p.i. for subsequent studies on CaM. We measured CaM level in presence of CMZ and BAPTA/AM. Pre-treatment with CMZ inhibited CaM expression (Fig 3B) implicating the role of CaM on *M*. *fortuitum-*induced HKM pathology. The inhibitory role of BAPTA/AM (Fig 3B) further proved CaM expression due to modulation of Ca^+2^ levels in the infected HKM.

The next step in this direction was to correlate CaM expression with HKM apoptosis. We observed that pre-treatment with CMZ attenuated caspase-3 activity (Fig 2C) and inhibited HKM apoptosis (Fig 2A and 2B) suggesting the role of CaM on *M*. *fortuitum* pathogenesis. Finally, we used CaM-siRNA to validate these results and observed that transfection with CaM-siRNA down-regulated CaM expression at mRNA (Fig 3C) and protein levels (Fig 3B) and attenuated *M*. *fortuitum*-induced HKM apoptosis (Fig 2A and 2B). Collectively, our results clearly establish the role of CaM in the cascade of events leading to *M*. *fortuitum*-induced HKM apoptosis.

### Activation of PKCα is critical for *M*. *fortuitum* pathogenesis

PKC is an important intermediate in Ca^+2^-signalling and implicated in apoptosis. Infection with *M*. *fortuitum* induced significant PKC activation with peak activity recorded 2 h p.i. (S3 Fig). Hence, this time interval was selected for subsequent PKC studies. The HKM were pre-treated with the pan-PKC inhibitor, CC and checked for enzyme activity and apoptosis respectively. The inhibitor binds with the catalytic domain of PKC and affects the translocation of the kinase from cytosol to plasma membrane [35]. CC down-regulated PKC activation (Fig 3D) and inhibited HKM apoptosis (Fig 2A and 2B) implicating PKC as pro-apoptotic molecule in *M*. *fortuitum* pathology. BAPTA/AM pre-treatment also down-regulated PKC activity (Fig 3D) confirming the involvement of Ca^+2^-dependent PKC in *M*. *fortuitum*-infected HKM.

Amongst the Ca^+2^-dependent PKC sub-family the role of PKCα has been reported in mycobacterial pathogenesis [10, 36, 37]. In this direction, the HKM were pre-treated with the PKCα inhibitor, Gö6976 and apoptosis assayed 24 h p.i. Gö6976 is a selective inhibitor of PKCα, though the mechanisms are not well understood [10, 11, 36]. It was observed that pre-treatment with Gö6976 down-regulated PKC activity (Fig 3D) and HKM apoptosis (Fig 2A and 2B). Based on these results we conclude that PKCα plays an important role in the pathogenicity of *M*. *fortuitum*.

### PKCα promotes superoxide production in *M*. *fortuitum*-infected HKM

**Fig 4:**
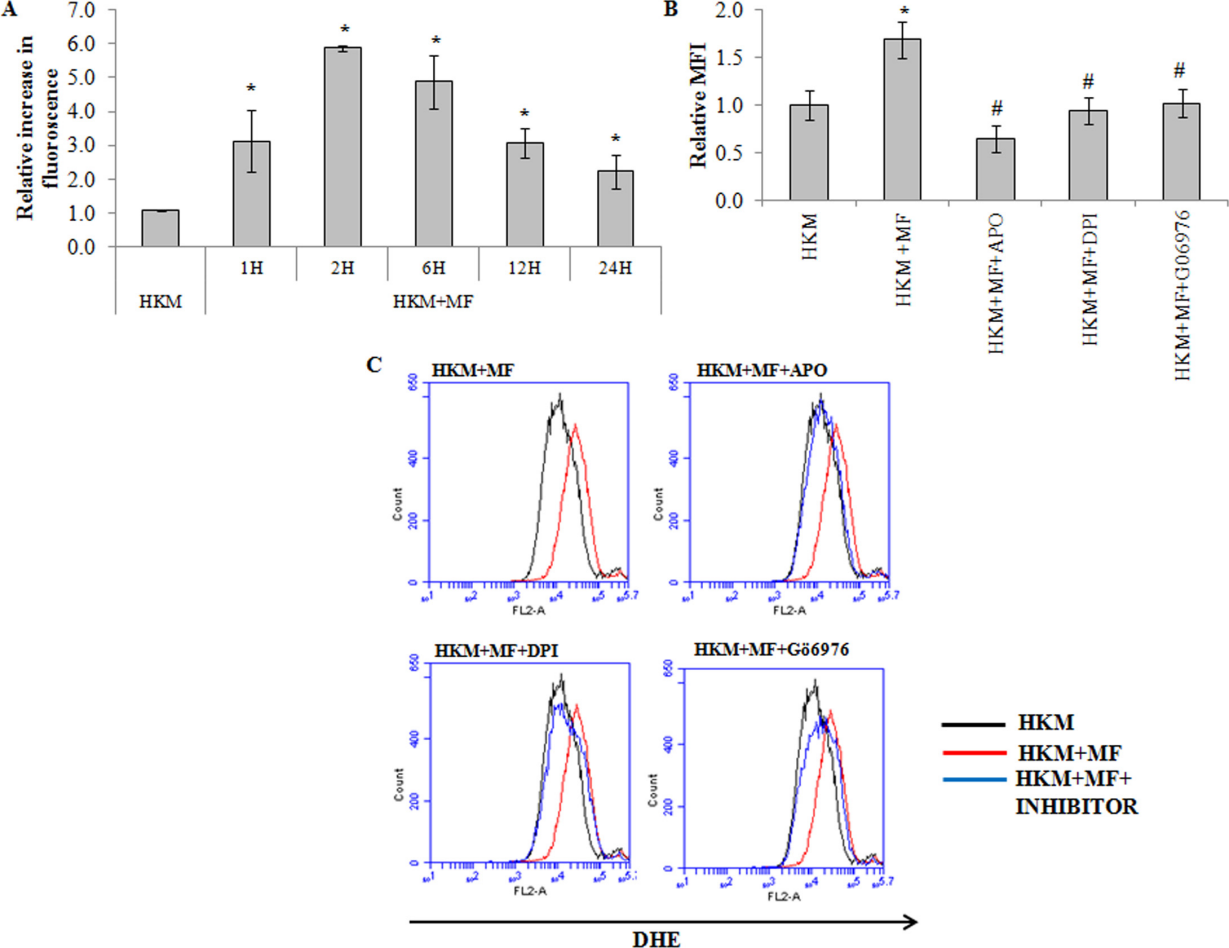
*M*. *fortuitum* induces PKCα-mediated superoxide generation. (A) HKM were infected with or without *M*. *fortuitum* and at indicated time p.i. superoxide generation measured using DHE. HKM were pre-treated separately with or without indicated inhibitors followed by *M*. *fortuitum* infection and superoxide generation measured 2 h p.i. by FACS using DHE (B) The relative median fluorescence intensity (MFI) and (C) Histogram. Vertical bars represent mean ± SE (n = 3).*P<0.05, compared to HKM; ^#^*P*<0.05, compared to HKM+MF. HKM, control head kidney macrophage; HKM+MF, HKM infected with *M*. *fortuitum;* HKM+MF+APO, HKM+MF+DPI, HKM+MF+Gö6976, HKM were pre treated with APO, DPI, and Gö6979 respectively followed by infection with *M*. *fortuitum*.

Reactive oxygen species (ROS) play a crucial role in host immunity to mycobacterial pathogens. However, the role of ROS in the pathogenesis of *M*. *fortuitum* is poorly understood. We used specific dye DHE and noted significant superoxide anion production in the *M*. *fortuitum*-infected HKM with maximum levels recorded 2 h p.i. though the levels remained significantly elevated till 24 h p.i. (Fig 4A). To correlate superoxide generation with *M*. *fortuitum* pathogenesis the HKM were pre-treated with antioxidants APO and DPI. Pre-treatment with APO and DPI down-regulated *M*. *fortuitum*-induced superoxide anion production (Fig 4B and 4C) and attenuated caspase-3 mediated apoptosis (Fig 2A, 2B and 2C) implicating the role of superoxide to the pathogenesis induced by the bacterium.

The next step was identifying the upstream events that lead to superoxide anion generation. PKCα dependent ROS production through the activation of NADPH Oxidase is reported earlier [11]. We hypothesised a similar role of PKCα in our study. To that end the HKM were pre-treated with Gö6976 and *M*. *fortuitum*-induced superoxide anion production studied. We observed that superoxide anion production was significantly reduced in presence of Gö6976 (Fig 4B and 4C) clearly indicating the pro-active role of PKCα on superoxide anion generation in *M*. *fortuitum*-infected HKM.

### CaM and superoxide converge to activate CaMKII*g* in *M*. *fortuitum* infected HKM

Calmodulin (CaM) induces its effects through several downstream kinases amongst which CaMKII is important. HKM were pre-treated separately with CaMKII specific inhibitor KN-93 and its structural analogue KN-92 and apoptosis assayed. KN-93 mediates its effect by competing for the CaM binding sites while KN-92 is the inactive analogue of KN-93, lacking CaM kinase inhibitory activity [8]. Pre-treatment with KN-93 attenuated caspase-3 activity and conferred significant protection to the infected HKM from undergoing apoptotic death (Fig 2A, 2B and 2C). Expectedly, HKM apoptosis could not be prevented in presence of KN-92, the inactive analogue of KN-93 suggesting a prime role of CaMKII on mediating *M*. *fortuitum*-induced HKM apoptosis.

**Fig 5:**
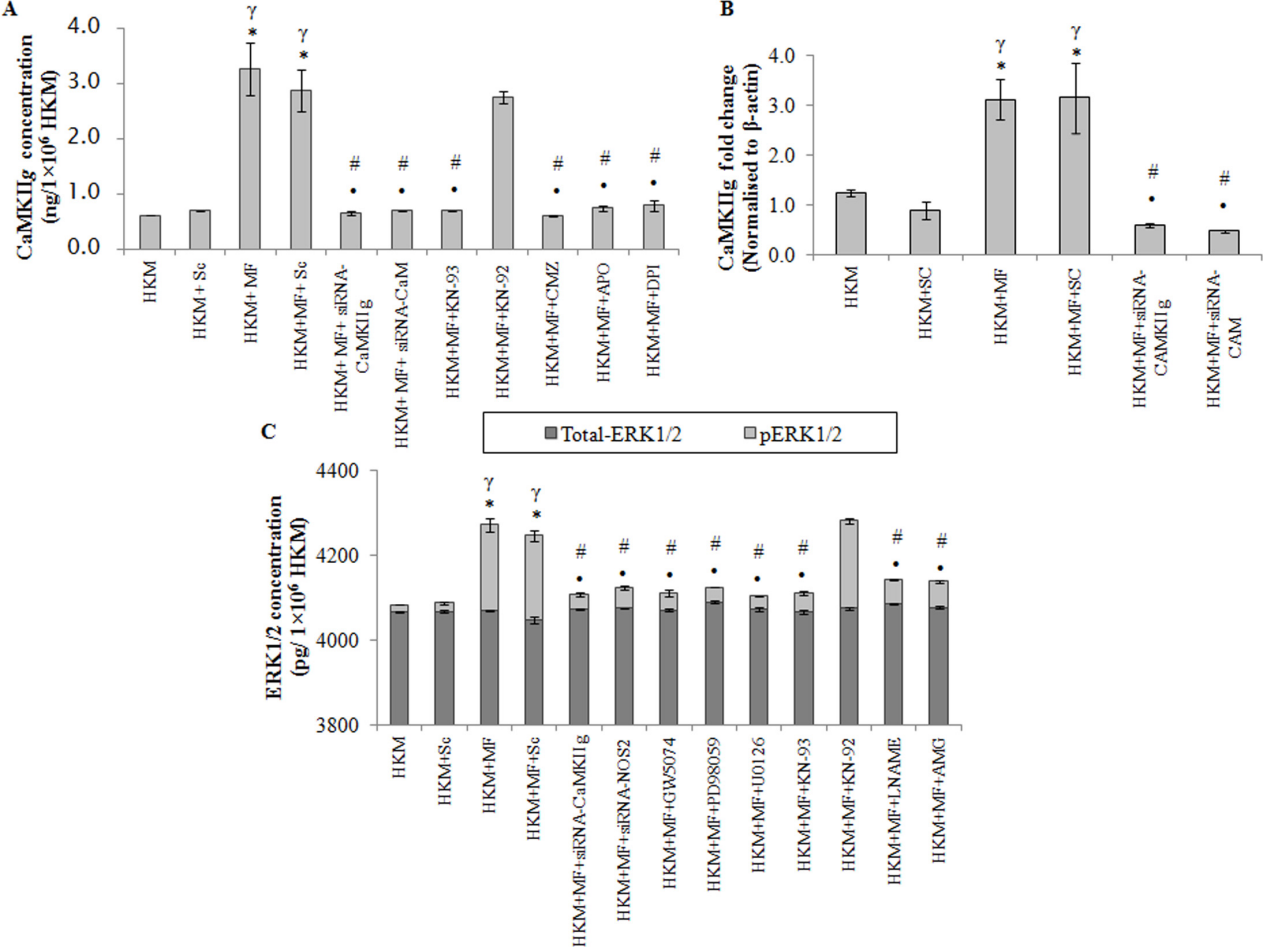
CaMKII*g* induces activation of pro-apoptotic ERK1/2 in *M*. *fortuitum* pathogenesis. HKM were transfected or pre-treated with or without indicated siRNAs or inhibitors respectively and CaMKII*g* expression determined by (A) EIA at 24 h p.i. and (B) qPCR at 4 h p.i. (C) HKM were transfected or pre-treated separately with or without indicated siRNAs or inhibitors respectively and at 24 h p.i. checked for total-ERK1/2 and pERK1/2 levels. Vertical bars represent mean ± SE (n = 3).*P<0.05, compared to HKM; ^γ^*P*<0.05, compared to HKM+Sc; ^#^*P*<0.05, compared to HKM+MF; ^•^*P*<0.05, compared to HKM+MF+Sc. HKM, control head kidney macrophage; HKM+Sc, HKM transfected with scrambled siRNA; HKM+MF, HKM infected with *M*. *fortuitum*; HKM+MF+Sc, HKM transfected with scrambled siRNA infected with *M*. *fortuitum*; HKM+MF+siRNA-CaM, HKM transfected with siRNA-CaM infected with *M*. *fortuitum*; HKM+MF+siRNA-CaMKII*g*, HKM transfected with siRNA-CaMKII*g* infected with *M*. *fortuitum*; HKM+MF+NOS2, HKM transfected with siRNA-NOS2 infected with *M*. *fortuitum*; HKM+MF+CMZ, HKM+MF+KN-93, HKM+MF+KN-92, HKM+MF+APO, HKM+MF+DPI, HKM+MF+GW5074, HKM+MF+PD98059, HKM+MF+U0126, HKM+MF+L-NAME, HKM+MF+AMG, HKM were pre treated with CMZ, KN-93, KN-92, APO, DPI, GW5074, PD98059, U0126, LNAME or AMG respectively followed by infection with *M*. *fortuitum*.

Among the several CaMKII isoforms, CaMKII*g* largely modulates macrophage functioning. The CaMKII*g* expression was checked by qPCR and results suggested maximum CaMKII*g*-mRNA expression 4 h p.i.; thereafter the levels though declined were significant till 24 h p.i. (S4 Fig). The next step was measuring CaMKII*g* level 24 h p.i. by EIA. We selected this time point because significant CaMKII*g* mRNA expression was observed even at 24 h p.i. Significant CaMKII*g* expression was observed in *M*. *fortuitum*-infected HKM which was inhibited in presence of KN-93 but not by KN-92 (Fig 5A). Pre-treatment with CMZ also suppressed CaMKII*g* level (Fig 5A).

Gene silencing was used to further validate our observations. We observed that transfection with CaM-and CaMKII*g*-siRNA down-regulated CaMKII*g* expression at mRNA (Fig 5B), protein level (Fig 5A) and attenuated *M*. *fortuitum*-induced HKM apoptosis (Fig 2A and 2B). Based on these observations we conclude that signalling via Ca^+2^-CaM-CaMKII*g* pathway is instrumental for initiating *M*. *fortuitum*-induced apoptotic cascade in HKM.

The NADPH Oxidase pathway has been reported to drive CaMKII activation following decline in Ca^+2^-CaM signals [38]. Our results with APO and DPI encouraged us to investigate this and we noted that CaMKII*g* activity was significantly inhibited in presence of APO and DPI (Fig 5A) suggesting the role of NADPH Oxidase/superoxide pathway on CaMKII*g* activation in *M*. *fortuitum*-infected HKM.

### ERK1/2 is pro-apoptotic and downstream to CaMKII*g*

ERK1/2 activation in mycobacterial pathogenesis is well established [14]. The activation of ERK1/2 depends on upstream Raf-1-MEK1/2 signalling. Hence, the HKM were infected with *M*. *fortuitum* in the presence of the pharmacological inhibitors GW5074, PD98059 and U0126 respectively and the changes in total-ERK1/2 and phosphorylated ERK1/2 (pERK1/2) levels measured 24 h p.i. (Fig 5C). GW5074 is a potent and selective Raf-1 inhibitor with no effect on the activity of MKK/MEK signalling. PD98059 is a non-ATP competitive inhibitor that binds to MKK/MEK1, preventing its activation by upstream protein kinases, such as Raf [39]; it does not directly inhibit ERK1/2. U0126, also a non-ATP competitive inhibitor prevents MEK from phosphorylating downstream ERK1/2 thereby inhibiting ERK1/2 functioning [8, 39]. *M*. *fortuitum* induced significant increase in pERK1/2 levels which was down-regulated in presence of GW5074, PD98059 and U0126 (Fig 5C). No noticeable change in total-ERK1/2 level was observed in the infected HKM.

The role of CaMKII on the activation of ERK1/2 has been reported in mycobacterial infection [7]. To address this HKM were pre-treated with KN-93 and KN-92, or transfected with CaMKII*g*-siRNA and *M*.*fortuitum*-induced changes in total-ERK1/2 and pERK1/2 levels studied 24 h p.i. To our expectation, KN-93 and CaMKII*g*-siRNA inhibited *M*. *fortuitum*-induced pERK1/2 expression but did not influence the total-ERK1/2 levels in infected or control HKM (Fig 5C).

Finally, the implication of ERK1/2 activation on *M*. *fortuitum*-infected HKM was studied. We observed that HKM apoptosis was attenuated in presence of GW5074, PD98059 and U0126 (Fig 2A and 2B). Based on these results we suggest that ERK1/2 is pro-apoptotic and downstream to CaMKII*g* in *M*. *fortuitum* infections.

### ERK1/2 and NOS2-NO crosstalk determines the fate of *M*. *fortuitum* infected HKM

**Fig 6:**
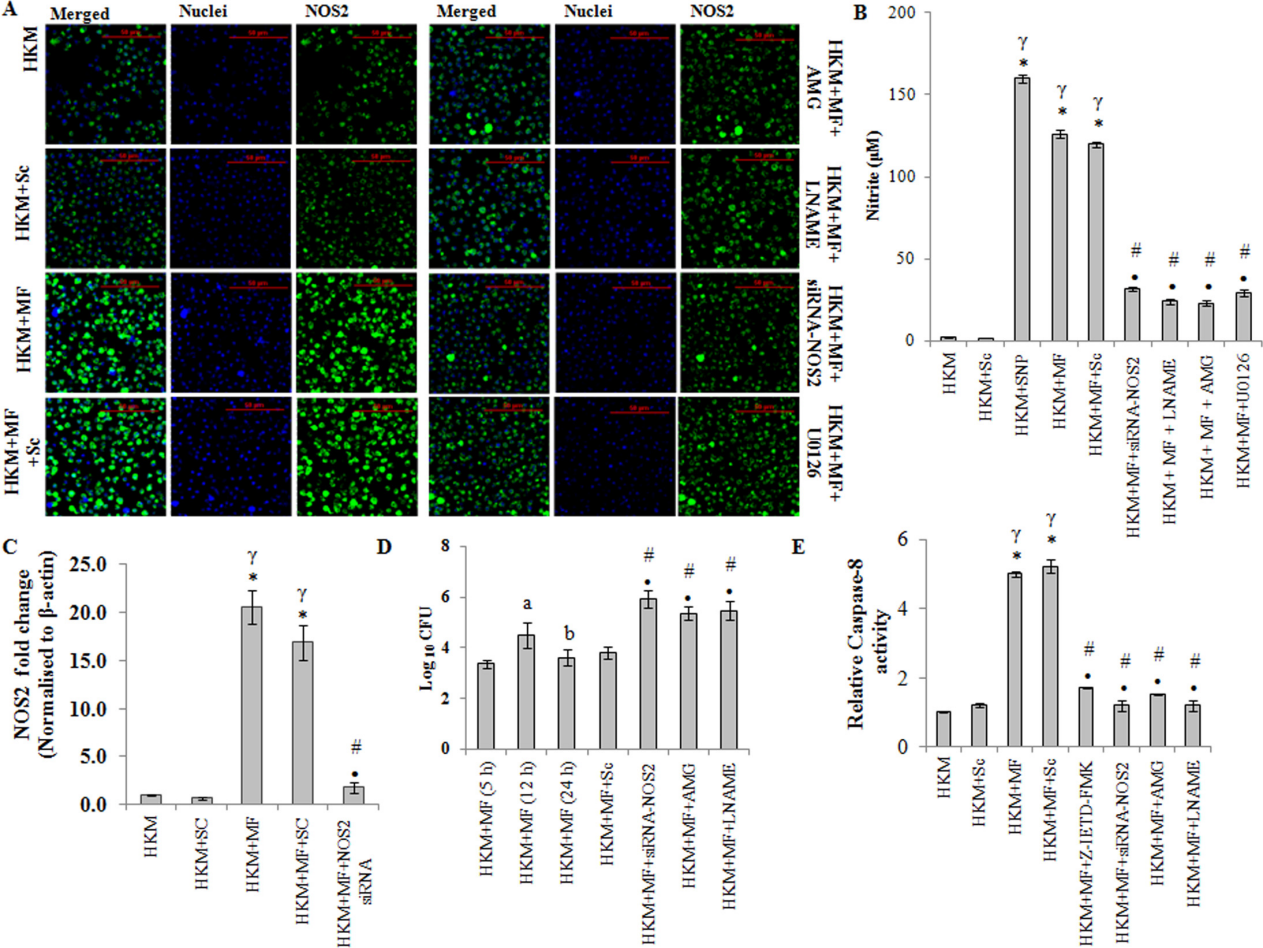
NOS2-NO axis induces caspase-8 activation in *M*. *fortuitum* infected HKM. (A) HKM were transfected or pre-treated separately with or without specific siRNA and indicated inhibitors respectively and at 24 h p.i. NOS2 protein (FITC conjugated) expression studied by confocal microscopy (×40). The images are representative of three independent experiments. (B) HKM were transfected or pre-treated separately with or without indicated siRNA or inhibitors respectively and at 24 h p.i. NO release measured. HKM treated with SNP was taken as positive control for the assay. (C) Fold change in NOS2 mRNA expression was determined in HKM transfected with specific siRNA or non targeted siRNA followed by *M*. *fortuitum* infection 12 h p.i. (D) Intracellular bacterial load was determined in HKM infected with *M*. *fortuitum* at indicated time p.i. or HKM transfected or pre-treated with indicated siRNA or inhibitors respectively 24 h p.i. by dilution plating on 7H11 agar plate. (E) Relative caspase-8 activity measured at 24 h p.i. in HKM pre-treated or transfected with indicated inhibitors or siRNA respectively. Vertical bars represent mean ± SE (n = 3).*P<0.05, compared to HKM; ^**γ**^*P*<0.05, compared to HKM+Sc; ^#^*P*<0.05, compared to HKM+MF; ^**•**^*P*<0.05, compared to HKM+MF+Sc; ^a^*P*<0.05, compared to HKM+MF (5 h); ^b^*P*<0.05, compared to HKM+MF (12 h). HKM, control head kidney macrophage; HKM+Sc, HKM transfected with scrambled siRNA; HKM+MF, HKM infected with *M*. *fortuitum*; HKM+MF+Sc, HKM transfected with scrambled siRNA infected with *M*. *fortuitum*; HKM+MF+siRNA-NOS2, HKM transfected with siRNA-NOS2 infected with *M*. *fortuitum*; HKM+MF+AMG, HKM+MF+LNAME, HKM+MF+U0126, HKM+MF+Z-IETD-FMK, HKM were pre-treated with AMG, LNAME, U0126 or Z-IETD-FMK, respectively followed by *M*. *fortuitum* infection.

Nitric oxide (NO) production in macrophages *via* NOS2 pathway is an important host defence mechanism against mycobacterial pathogens [17, 18]. However, the role of NO on *M*. *fortuitum-*induced pathogenicity is poorly understood. We observed significant NO production in HKM infected with *M*. *fortuitum* with maximum levels recorded 24 h p.i. (S4 Fig). We designed degenerate primers (Table 1) for NOS2 using the homologous stretch across vertebrates as the template and the PCR product was cloned and sequenced. The sequence showed 90% similarity with NOS2-mRNA of channel catfish, *Ictalurus punctatus*. Based on the sequence (Accession no. KF956810, Table 2) primers for qPCR studies were designed (Table 3) and NOS2-mRNA expression quantified. We observed maximum NOS2-mRNA expression at 12 h p.i. (S4 Fig) which declined at 24 h p.i. but was still significant in the infected HKM. The next step was to confirm NOS2 protein expression by immunofluroscence. As maximum NO production coupled with significant NOS2-mRNA expression was observed 24 h p.i. it was selected for subsequent studies. Our results clearly suggest increased NOS2 protein expression in *M*. *fortuitum* infected HKM (Fig 6A and S5 Fig).

Macrophages produce NO through the enzymatic conversion of L-arginine to L-citruline by NOS2. The inhibitors AMG and LNAME are structural analogues of L-arginine that prevent NOS2 activation. We observed that pre-treatment with AMG and LNAME (i) inhibited NOS2 expression (Fig 6A and S5 Fig), (ii) NO production (Fig 6B) and (iii) down-regulated caspase-3 activity and attenuated *M*. *fortuitum*-induced HKM apoptosis (Fig 2A, 2B and 2C). Sodium nitroprusside used as the positive control induced significant NO production in uninfected HKM. Together, these results suggested that induction of apoptosis is linked to NO production in *M*. *fortuitum-*infected HKM.

To further confirm this we used gene silencing approach. We noted that transfection with NOS2-siRNA down regulated NOS2 mRNA expression (Fig 6C), NOS2 protein expression (Fig 6A and S5 Fig), NO production (Fig 6B) and caspase-3 mediated HKM apoptosis (Fig 2A, 2B and 2C) which clearly established the importance of NOS2 dependent NO generation in *M*. *fortuitum*-induced HKM apoptosis.

Our next aim was studying the effect of NO on bacterial growth. The HKM were transfected or pre-treated separately with NOS2-siRNA or AMG and LNAME respectively and 24 h p.i. lysed, serially diluted and plated on 7H11 agar for enumerating CFU. It was noted that the survival of intra-cellular *M*. *fortuitum* was significantly improved by inactivating the NOS2-NO axis (Fig 6D) which confirmed the bactericidal role of NOS2-NO on *M*. *fortuitum*. The addition of AMG and LNAME in the culture medium did not influence bacterial growth *per se* (data not shown).

Once the importance of NOS2-NO axis on *M*. *fortuitum*-induced pathogenesis was established we looked for the upstream signals influencing NO release. ERK1/2 has been implicated on the transcriptional activation of NOS2 in mycobacterial pathogenesis [16]. There are also reports documenting the involvement of NO on the activation of ERK1/2 [40]. Hence, the HKM were pre-treated with U0126 to study NOS2 expression and NO production as well the impact of attenuating NOS2-NO signalling on ERK1/2 activation probed. We observed that pre-treatment with U0126 attenuated NOS2 expression (Fig 6A) and inhibited NO production (Fig 6B). Reversibly, attenuating the NOS2-NO signalling by AMG, LNAME or NOS2-siRNA inhibited the activation of ERK1/2 (Fig 5C). These results suggests a cross-talk between ERK1/2 and NOS2-NO axis in *M*. *fortuitum*-infected HKM.

### NO activates caspase-8 in *M*. *fortuitum* infected HKM

We sought to identify the apical caspases instigating the death process. Significant increase in caspase-8 activity was observed and pre-treatment with the caspase-8 inhibitor, Z-IETD-FMK attenuated its activity (Fig 6E) and reduced HKM apoptosis (Fig 2A and 2B). Concomitantly, Z-IETD-FMK also inhibited caspase-3 activity (Fig 2C) in the infected HKM. Based on these results we suggest the role of caspase-8 as initiator caspase in *M*. *fortuitum*-induced HKM apoptosis. Furthermore, transfection with NOS2-siRNA or pre-treatment with AMG or LNAME inhibited caspase-8 activation (Fig 6E). We conclude that NO induces it apoptotic influence by activating caspase-8 in *M*. *fortuitum*-infected HKM which activated caspase-3 to accomplish the apoptotic cascade.

## Discussion

There is little information on pathogenesis by atypical mycobacteria like *M*. *fortuitum*. Our results for the first time elucidate the hierarchy of pro-active signalling molecules in *M*. *fortuitum* pathogenesis.

We observed that *M*. *fortuitum* induces HKM apoptosis as evident by nuclear morphology, extra-membranous presence of phosphatidyl serine, host cell DNA fragmentation, development of cytoplasmic vacuoles and apoptotic bodies. Macrophagic death has been observed in presence of several intracellular pathogens including mycobacteria although the mechanisms and implications remain unexplained. *M*. *fortuitum*-induced apoptosis of human and murine macrophages has been reported earlier [41]. Our study for the first time reports the induction of similar pathological consequences in fish suggesting the pro-apoptotic trait to be a conserved virulence factor of *M*. *fortuitum* to counteract immune responses in different hosts.

Pathogen-induced intracellular Ca^+2^ imbalance affects cell survival and death [8, 42]. The role of Ca^+2^ signalling on mycobacterial pathogenesis is debatable with studies suggesting both pro- and anti-apoptotic effects on macrophages [4, 43]. From our results the pro-apoptotic involvement of Ca^+2^ in *M*. *fortuitum* infection was distinct. This is the first evidence linking increase in intra-cellular Ca^+2^ levels to *M*. *fortuitum*-induced macrophage apoptosis. The increase of Ca^+2^ levels are ‘deciphered’ by various intracellular Ca^+2^ binding proteins that convert the signals into a wide variety of biochemical changes. Among the several downstream Ca^+2^-binding proteins through which Ca^+2^ induces its varied effects CaM is important. It is one of the most conserved proteins and has also been reported from fish [8]. We found the significant role of CaM on *M*. *fortuitum* pathogenesis. Calmodulin showed parallel expression at transcription and translational level. This kind of observation has been noted with other earlier signalling molecules [44]. A similar trend of CaM expression has recently been reported from our laboratory in *A*. *hydrophila*-infected HKM [8]. Interestingly, we observed significant decline in CaM protein levels in presence of Ca^+2^ chelator BAPTA/AM as well as CMZ. How BAPTA/AM and CMZ influences CaM expression is not very clear to us. Earlier studies suggested the pro-active role of Ca^+2^ as second messenger in regulating the transcription of several proteins including CaM in plant [45] and mammalian cells [46]. Our results with BAPATA/AM, extends these observations suggesting a regulatory role of Ca^+2^ on the expression of CaM and CaM-dependent kinases in fish HKM. Regarding the role of CMZ on CaM expression there could be two possibilities. 1. CMZ regulates the cytosolic Ca^+2^ levels [47] thereby affecting the transcription of CaM. 2. It may contribute to off target actions that suppress CaM protein expression or stability. We propose that the increase in intracellular Ca^+2^ levels induced by *M*. *fortuitum* acts at two distinct levels. Firstly, at the molecular level it influences transcription and the mode of association of CaM with various downstream target proteins and secondly induces conformational states in CaM leading to target-specific activation and concomitant release of free energy critical for the transduction of Ca^+2^ signals in *M*. *fortuitum* infection.

There are several downstream targets for CaM amongst which the involvement of CaMKII is fairly well reported. CaMKII exists in several isoforms and their involvement in macrophage apoptosis is not well reported. We have used CaMKII*g* isoform for this study since it largely modulates macrophage functioning [8, 48]. Our findings clearly established the pro-apoptotic role of CaMKII*g* on *M*. *fortuitum*-pathogenesis. An interesting observation was the decrease in CaMKII*g* protein levels in presence of CMZ and KN-93 in the infected HKM. It is well known that activated-CaM can influence the transcription of several genes either directly or by modulating the activity of CaM-binding transcription factors (CAMTA) [45]. We hypothesize that the binding of CMZ compromises the transcriptional activity of CaM thereby affecting genes involved in the Ca^+2^-cascade like CaMKII. The fluctuations in cytosolic Ca^+2^ levels also influence CaMKII transcription [46]. CMZ and KN-93, besides inhibiting intra-cellular Ca^+2^ levels also interferes with cyclic nucleotide metabolism and G protein mediated signalling important for regulating the transcriptional activity of CaMKII [47, 49]. Increased CaMKII expression was reported earlier in *M*. *bovis* [50] and *M*. *smegmatis* [7], and our results extend this to *M*. *fortuitum* suggesting CaMKII*g* as a virulence factor in host though the mechanisms remain to be established. In this context identifying the role of other CaMKII isoforms in *M*. *fortuitum*-pathogenesis would be interesting.

Members of the PKC family have been implicated in mycobacterial pathogenesis but their involvement during *M*. *fortuitum* infections remains to be addressed. Our data clearly suggested the pro-apoptotic role of PKCα on *M*. *fortuitum-*pathology. Earlier studies suggested PKCα to be crucial for *M*. *bovis* BCG [36] and *M*. *tuberculosis* [37]. Thus, our results confirm earlier observations reporting PKCα modulation to be a conserved strategy of mycobacterial pathogens to induce pathological changes.

ROS produced by macrophages induces microbicidal effects. However, ROS generation in response to mycobacterial infections has been a contentious issue. There are reports suggesting mycobacteria-induced ROS generation to be pro-apoptotic hence a critical host resistance determinant [12]. It has also been proposed that mycobacteria have evolved mechanism to impair or detoxify ROS to facilitate intracellular survival [51]. The NADPH oxidase complex is well characterised and correlated with superoxide generation in fish macrophages against mycobacterial pathogens [52]. We observed increased superoxide generation consequent to *M*. *fortuitum* infection and pre-treatment with APO and DPI significantly reduced superoxide generation and HKM apoptosis. These results are in line with earlier studies reporting the role of *M*. *fortuitum*-induced robust ROS generation in mammalian macrophage apoptosis [53]. The role of PKC on ROS generation through the activation of NADPH Oxidase has been demonstrated under various conditions [11]. Only recently, the involvement of different PKC isoforms on ROS generation is becoming apparent. We observed that inhibiting PKCα suppressed superoxide generation implicating its role on ROS generation in *M*. *fortuitum* infection. We postulate PKCα might be involved in the phosphorylation and membrane translocation of the enzyme components [54].

An unexpected and intriguing observation we made is that significant CaMKII*g* activity was recorded at late hours of infection when both Ca^+2^ and CaM levels had declined significantly. Coupled to that we noted sustained ROS generation in *M*. *fortuitum* infected HKM. Each CaMKII monomer consists of an N-terminal catalytic domain a C-terminal association domain that enables assembly of the holoenzyme and a regulatory domain between the catalytic and association domains. Under conditions of brief increases in intracellular Ca^+2^ levels CaMKII returns to an inactive conformation after Ca^+2^/CaM unbinding. Only recently it was observed that ROS can prolong CaMKII activation in absence of Ca^+2^/CaM signaling *via* oxidation of methionine residues present in the regulatory subunit [38]. There are no reports on this alternate mechanism of CaMKII activation in microbe-induced pathogenesis. We observed that ROS induced via PKCα contributes to CaMKII*g* activation in the HKM in absence of Ca^+2^-CaM signalling. Our results support the mechanism by which CaMKII can integrate Ca^+2^ and ROS signals [38]. We propose that the Ca^+2^/CaM binding serves as the initial trigger for CaMKII activation in *M*. *fortuitum* pathogenesis. Subsequently, the pro-oxidant rich conditions in the infected HKM induce the oxidation of methionine residues in CaMKII which resets the Ca^+2^ sensitivity of CaMKII so that very low levels of intracellular Ca^+2^ are sufficient for prolonging the kinase activity. The constutitively active Ca^+2^/CaM autonomous CaMKII promotes core events important for initiating the apoptotic cascade in *M*. *fortuitum* infected HKM. It is important to note that oxidized CaMKII is reduced and inactivated by methionine sulfoxide reductase A (MsrA) [5]. We believe that following *M*. *fortuitum* infections the balance between oxidised CaMKII and Met-reduced CaMKII is lost in the HKM. Thus, understanding of the ox-CaMKII/MsrA signaling pathway would provide new insights into how Ca^+2^/CaM-ROS-CaMKII axis causes apoptosis of *M*. *fortuitum* infected HKM.

The activation of ERK1/2 plays a critical role in microbial pathogenesis. However, there is little evidence suggesting ERK1/2 activation by *M*. *fortuitum*. The role of ERK1/2 as pro-apoptotic molecule has been demonstrated against *M*. *avium* [15] and *M*. *bovis* BCG [6]. Based on our results and earlier reports we suggest that ERK1/2 activation is an important virulence mechanism in *M*. *fortuitum* pathogenesis. Earlier studies have also indicated the role of CaMKII on activating the ERK1/2 pathway [7]. We observed that inhibiting CaMKII*g* down-regulated ERK1/2 activation which confirmed that ERK1/2 is downstream to Ca^+2^-CaM-CaMKII*g* signalling in *M*. *fortuitum*-infected HKM. How CaMKII*g* activates ERK1/2 was not investigated by us. However, it was earlier observed that CaMKII can bind to Raf to initiate the activation of ERK1/2 [55]. We observed that PD98059 is also a Raf inhibitor [39] down regulated ERK1/2 phosphorylation suggesting that ERK1/2 activation by CaMKII*g* occurs *via* Raf kinase. Amongst the different Raf isoforms, Raf-1 is the upstream regulator of the MEK-ERK1/2 pathway and its presence was reported in fish [56]. For a direct proof we assayed ERK1/2 in presence of the Raf-1 inhibitor, GW5074, and our results proved that Raf-1 plays the role of upstream regulator in CaMKII*g* induced ERK1/2 activation. Thus we conclude that CaMKII*g* triggered Raf-1-MEK1/2-ERK1/2 signalling during *M*. *fortuitum* infection.

NOS2-induced NO plays an important role in host defence against microbial pathogens [17, 18]. It appears that NO act as pro-and anti-apoptotic factor depending upon experimental conditions [57]. Using a combination of specific inhibitors and specific siRNA we report that NO helps in the containment of the intra-cellular bacteria and acts as pro-apoptotic molecule in *M*. *fortuitum* pathology. Our findings contradict earlier studies reporting *M*. *fortuitum* does not produce NO in mammalian macrophages [18]. The ability of mycobacteria to survive and induce pathology varies among different host species [52, 58]. Fish is the natural host for *M*. *fortuitum* which could be the reason behind the observed differences with mammalian macrophages.

The role of NO on piscine mycobacteriosis has not been studied in details. In context of the *M*. *marinum*-goldfish and -zebrafish models a decline in NO production was reported which aided in the spread and persistence of the bacterium [52, 58]. Looking at these contradictory results it seems likely that the different fish pathogenic mycobacteria alike the mammalian counterparts have evolved multiple mechanisms for countering macrophagic responses in different hosts.

Signalling through MAPK pathway influences NO production during mycobacterial infections [16]. We asked whether ERK1/2 has a role on NO production in *M*. *fortuitum* infected HKM. Our inhibitor studies clearly demonstrated *M*. *fortuitum* induced NO production is linked to MEK1/2-ERK1/2 signalling. These results are in line with earlier studies suggesting a role of ERK1/2 on inducing NO release in macrophages infected with mycobacterial pathogens [16]. It has been proposed that ERK1/2 could modulate NOS2 *via* release of pro-inflammatory cytokines like TNF-α [59] with NFκB playing an intermediatory role in the process [60, 61]. Further studies are needed to identify the role of other macrophage soluble mediators like TNF-α, IL-1β as well as NFκB and co-relate them with NO-mediated HKM apoptosis in our model. There are several recent reports implicating NO in the activation of ERK1/2 with pathological consequences [40]. To the best of our knowledge this has not been reported during host-mycobacterial interactions. This prompted us to examine the interaction between NO and ERK1/2 in *M*. *fortuitum*-infected HKM. We noted that inhibiting NO production led to down-regulation of ERK1/2 activation. It has been reported that NO activates ERK1/2 pathway in cGMP/PKG-dependent as well as Ras-dependent but cGMP- independent [40] manner. These mechanisms may be involved in the activation of ERK1/2 by NO in *M*. *fortuitum*-infected HKM. Our results for the first time demonstrated ERK1/2-NO cross-talk in mycobacterial pathogenesis paving way for understanding of the molecular mechanisms of mycobacteriosis. It is important to note that ERK1/2 and NO-mediated effects depend on the duration of exposure and their levels in the cells [40]. We propose that a positive feedback mechanism operates between ERK1/2 and NO due to which their levels increase significantly and once it crosses the critical threshold it tilts the balance in favour of apoptosis.

The involvement of initiator caspase-8 and caspase-9 has been observed in mycobacterial pathogenesis [21] but their role in *M*. *fortuitum-*pathogenesis is unknown. In the present work we focussed on caspase-8 which initiates extrinsic pathway of apoptosis. Our results clearly implicated caspase-8 involvement on initiating the *M*. *fortuitum*-induced apoptotic cascade in HKM. We noted that inhibiting the NOS2-NO axis by inhibitors and gene silencing approach significantly reduced caspase-8 activity and provided significant protection to HKM from undergoing apoptosis. Our results proved that NO contributes towards the activation of caspase-8 in *M*. *fortuitum* infected HKM. How NO activates caspase-8 is not clear to us and further studies are needed to understand this cross-talk. We extended our study by showing that consequent to NOS2-NO inhibition caspase-3 activation was abrogated. We conclude that caspase-8 acts as conduit between NO and caspase-3 in *M*. *fortuitum-*infected HKM. Based on our observations we suggest *M*. *fortuitum* induces HKM apoptosis through a caspase 8-dependent pathway and NO promotes apoptosis by amplifying caspase-8 and -3 activation. In this context it would be interesting to investigate the involvement of caspase-9 and study the cross-talk between the two initiator caspases during *M*. *fortuitum* infection.

**Fig 7:**
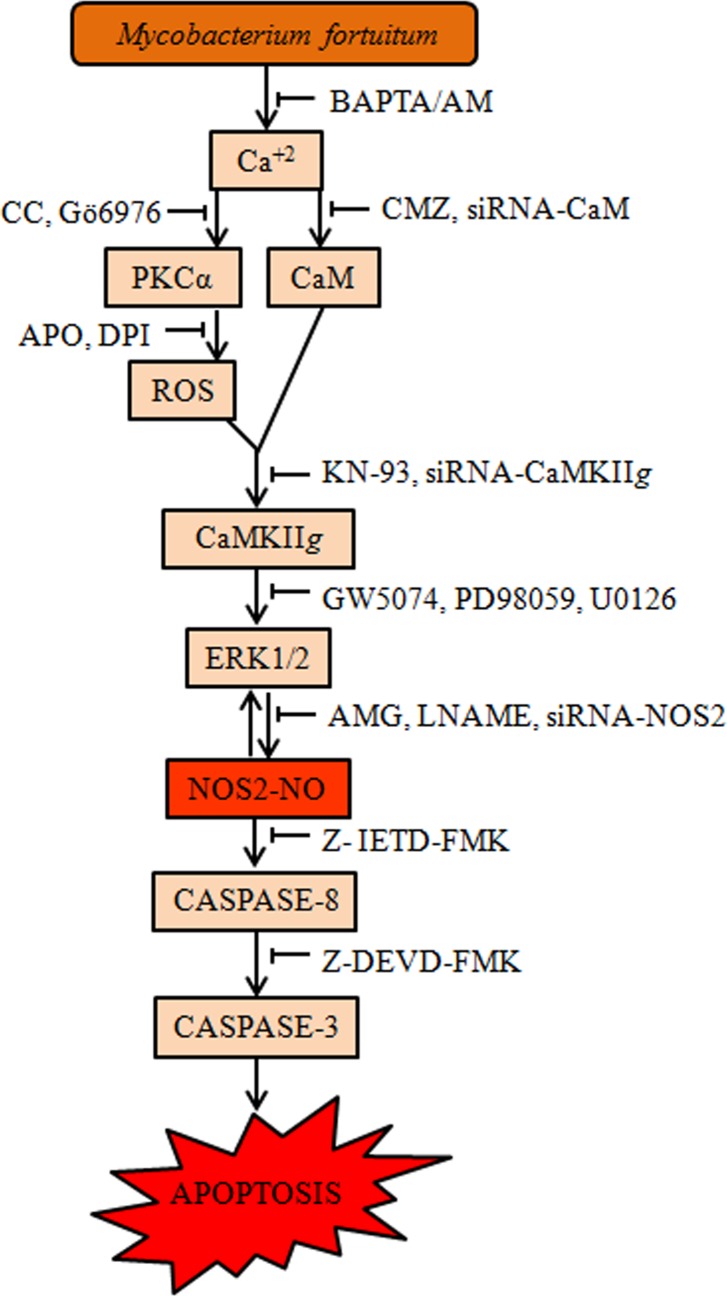
Overview of the work. *M*. *fortuitum* alters intracellular Ca^+2^homeostasis leading to activation of CaM and PKCα. PKCα induces superoxide anions generation *via* NADPH Oxidase. CaM and superoxide together activates CaMKII*g*. In downstream, CaMKII*g* modulates ERK1/2 to activate NOS2-NO axis. The ensuing ERK1/2-NO positive feedback loop leads to caspase-8 mediated caspase-3 activation and HKM apoptosis.

Our study for the first time traces the course of events that lead to *M*. *fortuitum* induced macrophage apoptosis in fish (Fig 7). We propose that *M*. *fortuitum* infection alters the intracellular Ca^+2^ levels. CaM and the robust superoxide levels induced via-PKCα-NADPH Oxidase converges to prolong CaMKII*g* activations thereby sustaining the levels of ERK1/2 critical for NO release. The cross-talk between ERK1/2 and NO ‘places checks and balances’ eventually tilting in favour of caspase activation and HKM apoptosis. We are currently identifying which mycobacterial molecule (s) mediate this signalling cascade and if specifically modifying this apoptotic programme changes the course of infection *in vivo*.

## Supporting Information

S1 Fig *M*. *fortuitum* induces HKM apoptosis. (PDF)

S2 Fig Inhibition of Ca^+2^ and caspase activity enhanced the intracellular bacterial load. (PDF)

S3 Fig *M*. *fortuitum* increases cytosolic Ca^+2^ leading to the activation of CaM and PKC. (PDF)

S4 Fig *M*. *fortuitum* induces the over expression of CaMKII*g* and NOS2/NO in infected HKM. (PDF)

S5 Fig *M*. *fortuitum* induces NOS2 activation in infected HKM. (PDF)
