# Supplementary material for: Calcium and Superoxide-Mediated Pathways Converge to Induce Nitric Oxide-Dependent Apoptosis in Mycobacterium fortuitum-Infected Fish Macrophages
Source: PLoS One. 2016 Jan 11;11(1):e0146554. doi: 10.1371/journal.pone.0146554 (PMC4713470; doi:10.1371/journal.pone.0146554)
Supplement: S1 Fig — (PDF) [file pone.0146554.s001.pdf]

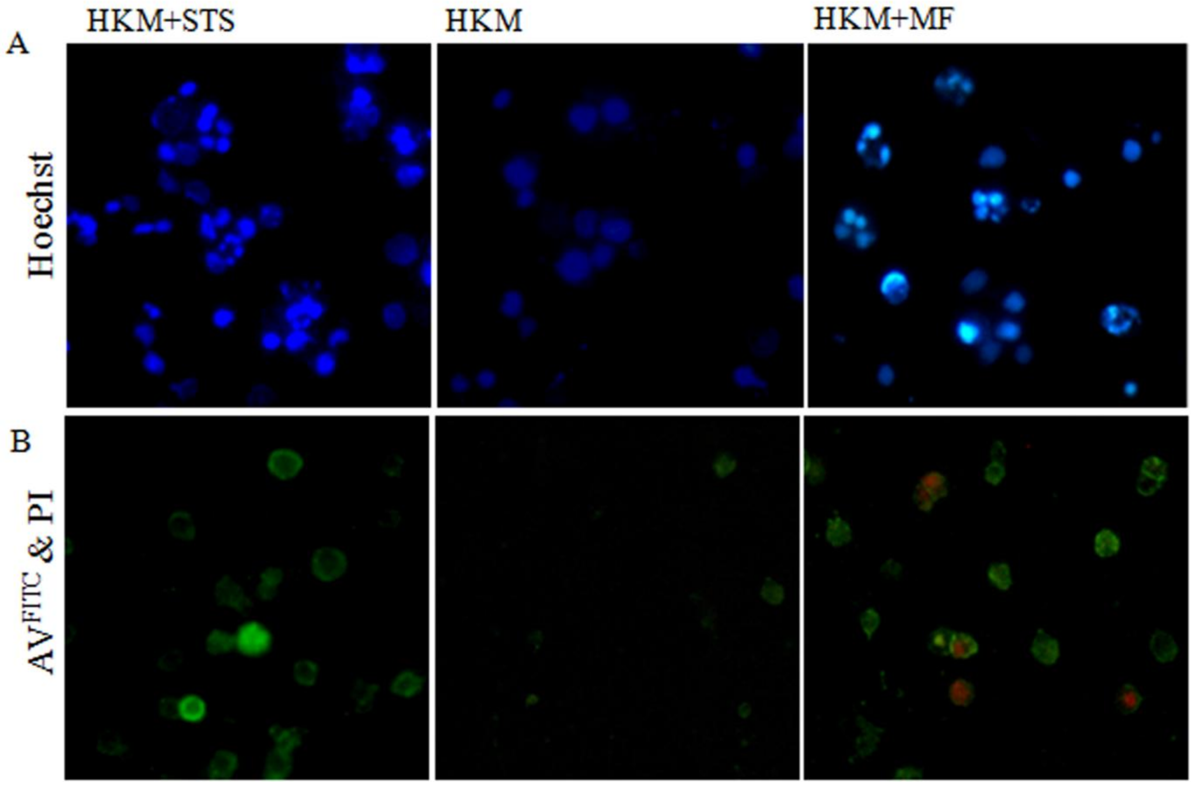

S1 Fig. *M. fortuitum* induces HKM apoptosis.

(A) Hoechst staining of STS treated (positive control), uninfected and *M. fortuitum*-infected HKM. (B) AV^{FITC}/PI staining of STS treated (positive control), uninfected and *M. fortuitum*-infected HKM. The images were observed under fluorescence microscope (×40). All the experiments were performed 24 h p.i. The images are representative of three independent experiments.
