# Supplementary material for: Calcium and Superoxide-Mediated Pathways Converge to Induce Nitric Oxide-Dependent Apoptosis in Mycobacterium fortuitum-Infected Fish Macrophages
Source: PLoS One. 2016 Jan 11;11(1):e0146554. doi: 10.1371/journal.pone.0146554 (PMC4713470; doi:10.1371/journal.pone.0146554)
Supplement: S2 Fig — (PDF) [file pone.0146554.s002.pdf]

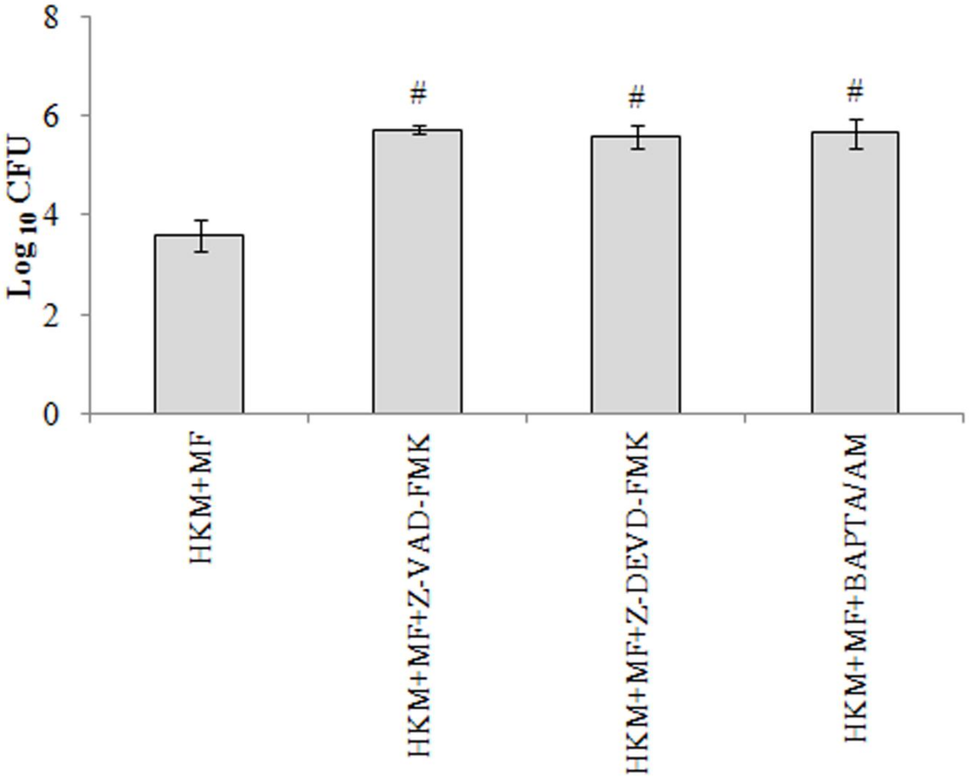

S2 Fig. Inhibition of Ca^{+2} and caspase activity enhanced the intracellular bacterial load.

Intracellular bacterial replication was quantified by dilution plating of HKM pre-treated with indicated inhibitors followed by *M. fortuitum* infection 24 h p.i. Vertical bars represent mean ± SE (n=3). $^{\#}P<0.05$, compared to HKM+MF; HKM+MF, HKM infected with *M. fortuitum*; HKM+MF+Z-VAD-FMK, HKM+MF+Z-DEVD-FMK, HKM, HKM+MF+BAPTA/AM, HKM were pre-treated with Z-VAD-FMK, Z-DEVD-FMK and BAPTA/AM respectively followed by *M. fortuitum* infection.
