# Supplementary material for: Calcium and Superoxide-Mediated Pathways Converge to Induce Nitric Oxide-Dependent Apoptosis in Mycobacterium fortuitum-Infected Fish Macrophages
Source: PLoS One. 2016 Jan 11;11(1):e0146554. doi: 10.1371/journal.pone.0146554 (PMC4713470; doi:10.1371/journal.pone.0146554)
Supplement: S3 Fig — (PDF) [file pone.0146554.s003.pdf]

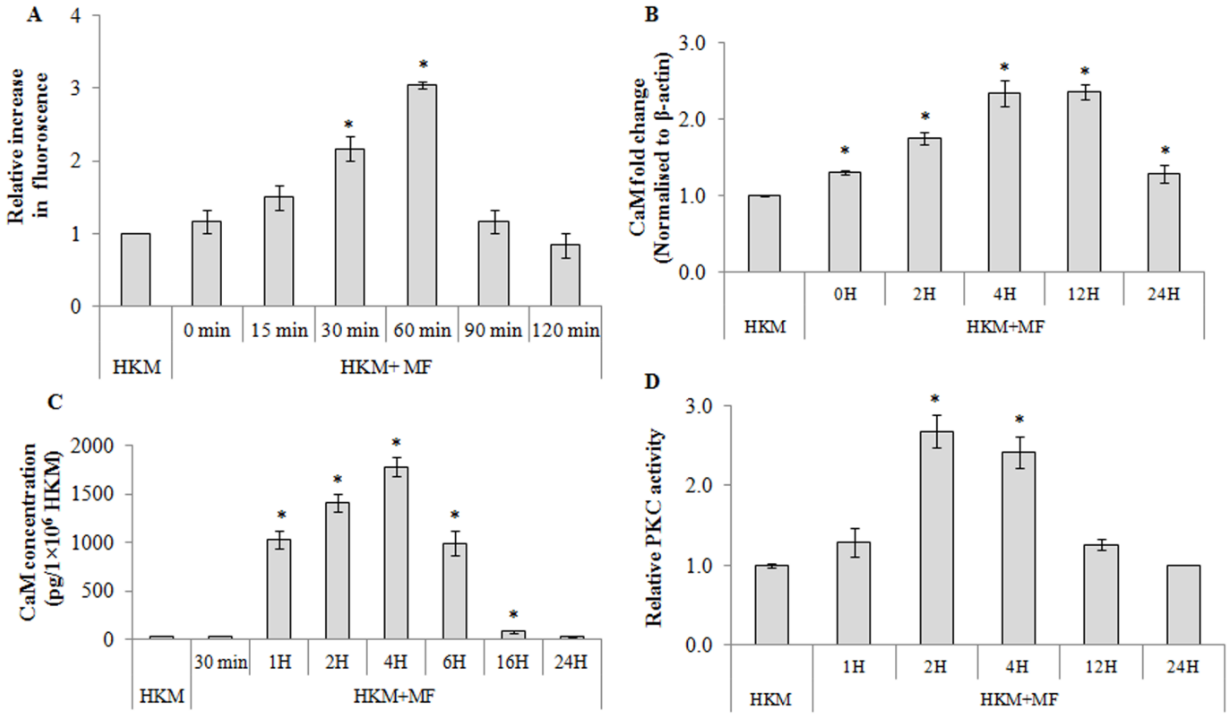

S3 Fig. *M. fortuitum* increases cytosolic Ca^{+2} leading to the activation of CaM and PKC.

(A) HKM uninfected or infected with *M. fortuitum* and at indicated time of infection the cytosolic Ca^{+2} level was quantified using Fluo-4/AM. HKM uninfected or infected with *M. fortuitum* and at indicated time p.i. the CaM espression was measured at (B) mRNA level and (C) protein level. (D) HKM uninfected or infected with *M. fortuitum* and at indicated time p.i. PKC activity was measured. Vertical bars represent mean ± SE (n=3).*P<0.05, compared to HKM. HKM, control head kidney macrophage; HKM+MF, HKM infected with *M. fortuitum*.
