# Supplementary material for: Calcium and Superoxide-Mediated Pathways Converge to Induce Nitric Oxide-Dependent Apoptosis in Mycobacterium fortuitum-Infected Fish Macrophages
Source: PLoS One. 2016 Jan 11;11(1):e0146554. doi: 10.1371/journal.pone.0146554 (PMC4713470; doi:10.1371/journal.pone.0146554)
Supplement: S4 Fig — (PDF) [file pone.0146554.s004.pdf]

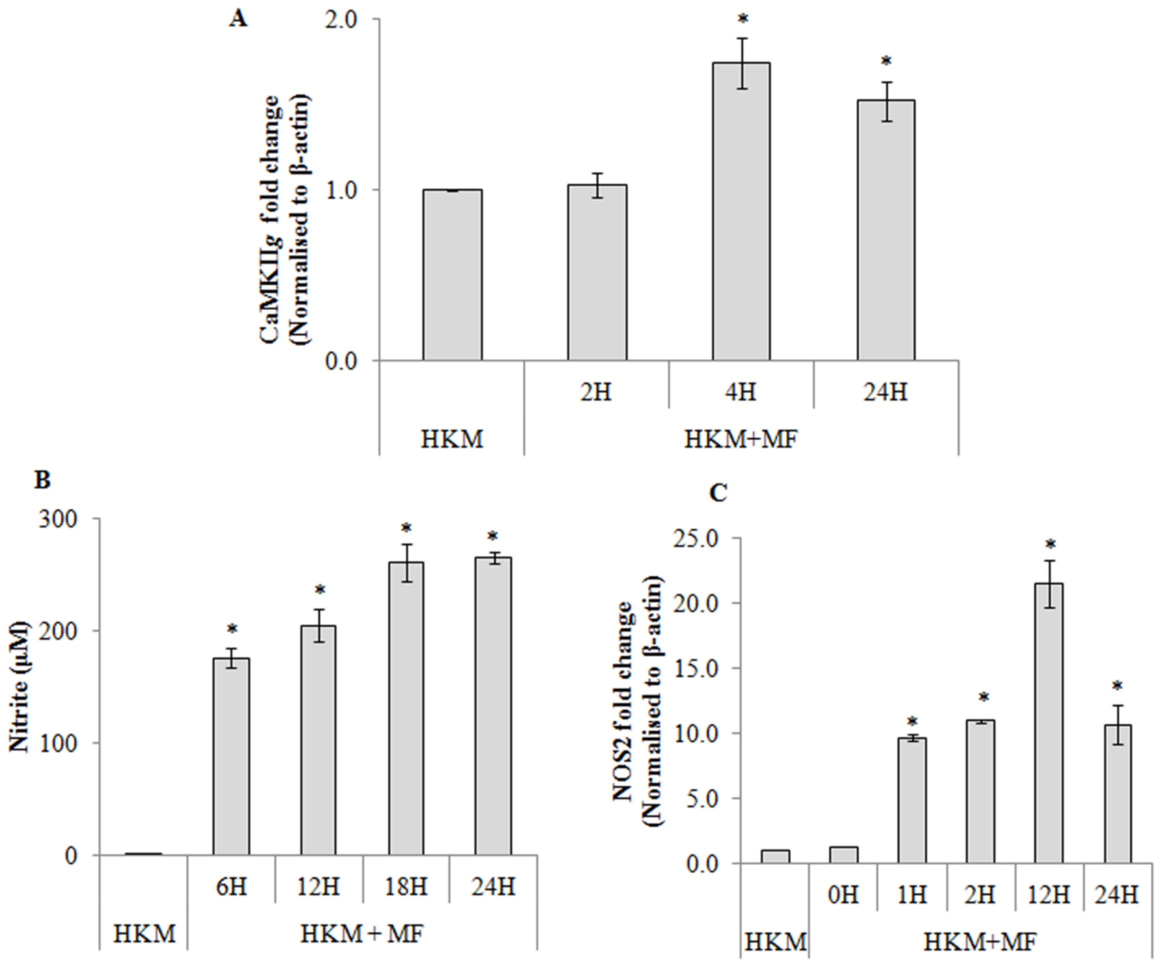


## S4 Fig. *M. fortuitum* induces the over-expression of CaMKII*g* and NOS2/NO in infected HKM

HKM uninfected or infected with *M. fortuitum* and at indicated time p.i. (A) CaMKII*g* mRNA expression (B) NO release and (C) NOS2 mRNA expression were measured. Vertical bars represent mean ± SE (n=3).*$P$<0.05, compared to HKM. HKM, control head kidney macrophage; HKM+MF, HKM infected with *M. fortuitum*.
