# Supplementary material for: Calcium and Superoxide-Mediated Pathways Converge to Induce Nitric Oxide-Dependent Apoptosis in Mycobacterium fortuitum-Infected Fish Macrophages
Source: PLoS One. 2016 Jan 11;11(1):e0146554. doi: 10.1371/journal.pone.0146554 (PMC4713470; doi:10.1371/journal.pone.0146554)
Supplement: S5 Fig — (PDF) [file pone.0146554.s005.pdf]

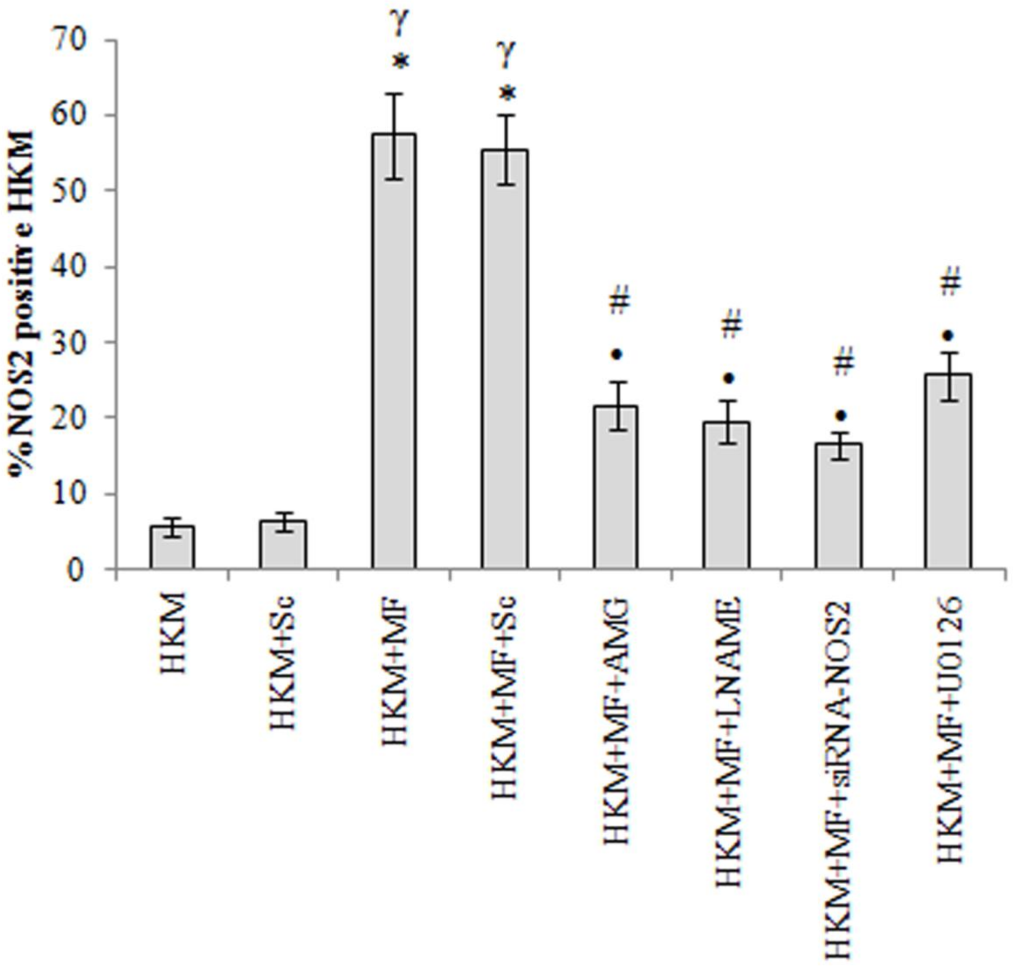

S5 Fig. *M. fortuitum* induces NOS2 activation in infected HKM

HKM were transfected or pre-treated separately with or without specific siRNA and indicated inhibitors respectively and at 24 h p.i. NOS2 protein expression studied by confocal microscopy (×40). The percent NOS2 positive cells were plotted. Hundred HKM were studied in each field and three such fields were included to determine the percentage of NOS2 positive HKM. Vertical bars represent mean ± SE (n=3).*P<0.05, compared to HKM; $^{\gamma}P$<0.05, compared to HKM+Sc; $^{\#}P$<0.05, compared to HKM+MF; $^{\bullet}P$<0.05, compared to HKM+MF+Sc. HKM, control head kidney macrophage; HKM+Sc, HKM transfected with scrambled siRNA; HKM+MF, HKM infected with *M. fortuitum*; HKM+MF+Sc, HKM

transfected with scrambled siRNA infected with *M. fortuitum*; HKM+MF+siRNA-NOS2, HKM transfected with siRNA-NOS2 infected with *M. fortuitum*; HKM+MF+AMG, HKM+MF+LNAME, HKM+MF+U0126, HKM+MF+Z-IETD-FMK, HKM were pre-treated with AMG, LNAME, U0126 or Z-IETD-FMK, respectively followed by *M. fortuitum* infection.
